# Immunomodulatory Effects and Regulatory Mechanisms of (*R*)-6-HITC, an Isothiocyanate from Wasabi (*Eutrema japonicum*), in an *Ex Vivo* Mouse Model of LPS-Induced Inflammation

**DOI:** 10.1021/acs.jafc.4c02943

**Published:** 2024-09-19

**Authors:** Manuel Alcarranza, Catalina Alarcón-de-la-Lastra, Rocío Recio Jiménez, Inmaculada Fernández, María Luisa Castejón Martínez, Isabel Villegas

**Affiliations:** † Instituto de Biomedicina de Sevilla, IBiS/Hospital Universitario Virgen del Rocío/CSIC/Universidad de Sevilla, 41013 Sevilla, Spain; ‡ Departamento de Farmacología, Facultad de Farmacia, Universidad de Sevilla, 41012 Sevilla, Spain; § Departamento de Química Orgánica y Farmacéutica, Facultad de Farmacia, Universidad de Sevilla, 41012 Sevilla, Spain

**Keywords:** (R)-6-HITC, DAG-methodology, murine peritoneal macrophages, inflammation, Eutrema japonicum, wasabi

## Abstract

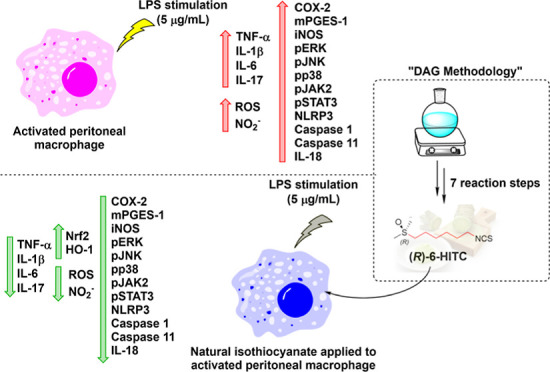

The present study aimed to investigate the effects of (*R*)-(−)-1-isothiocyanato-6-(methylsulfinyl)-hexane [(*R*)-6-HITC], the major isothiocyanate present in wasabi, in an *ex vivo* model of inflammation using lipopolysaccharide-stimulated murine peritoneal macrophages. (*R*)-6-HITC improved the immune response and mitigated oxidative stress, which involved suppression of reactive oxygen species, nitric oxide, and pro-inflammatory cytokines (IL-1β, IL-6, IL-17, IL-18, and TNF-α) production and downregulation of pro-inflammatory enzymes such as inducible nitric oxide synthase, COX-2, and mPGES-1. In addition, (*R*)-6-HITC was able to activate the Nrf2/HO-1 axis while simultaneously inhibiting key signaling pathways, including JAK2/STAT3, mitogen-activated protein kinases, and canonical and noncanonical inflammasome pathways, orchestrating its potent immunomodulatory effects. Collectively, these findings demonstrate the potential of (*R*)-6-HITC as a promising nutraceutical for the management of immuno-inflammatory diseases and justify the need for further *in vivo* validation studies.

## 1 Introduction

*Eutrema japonicum* (*Eutrema wasabi* or *Wasabia japonica* Matsum) also known as Japanese horseradish, is one of the most important species of Cruciferae, belonging to the Brassicaceae family, including broccoli, rocket, cauliflower and watercress.^1^ Although the whole plant is edible, only the fleshy rhizomes are used to prepare a famous green sauce with a pasty consistency from their grating.^2^ The resulting sauce is wasabi, an essential ingredient to serve as an accompaniment to typical food in Japan, which gives the popular name to the plant of provenance.^3^

In addition to its consumption as a condiment, wasabi has been used as a folk remedy for various disorders. Recent in vitro assays have shown interesting bioactive effects, including cytotoxic,^4,5^ anti-inflammatory,^4,6^ and antimicrobial in human colon adenocarcinoma cells and human oral epithelial cells.^4,7,8^ In in vivo studies, wasabi has shown antiobesity and antihypertensive effects in rats^9^ and neuroprotective effects against Parkinson’s disease in mice.^10^ Although few clinical trials have been conducted on wasabi consumption, some of them highlight its therapeutic role against inflammation generated in myalgic encephalomyelitis/chronic fatigue syndrome.^11^ Additionally, it has been observed that the consumption of wasabi leaf extract exhibited antiaging, antioxidant, antiglycating, and whitening effects.^12^

In general, wasabi is low in calories, contains high amounts of water, is rich in fiber, vitamins and minerals, and is an excellent source of secondary metabolites, including glucosinolates (GLSs), phenolic compounds, triterpenes, tocopherol, and carotenoids, among others.^13,14^

The main bioactive degradation products of GLSs are isothiocyanates (ITCs). Wasabi has high levels of long-chain ITCs such as 6-methylsulfinylhexyl ITC (6-HITC).^15,16^

**Scheme 1:**
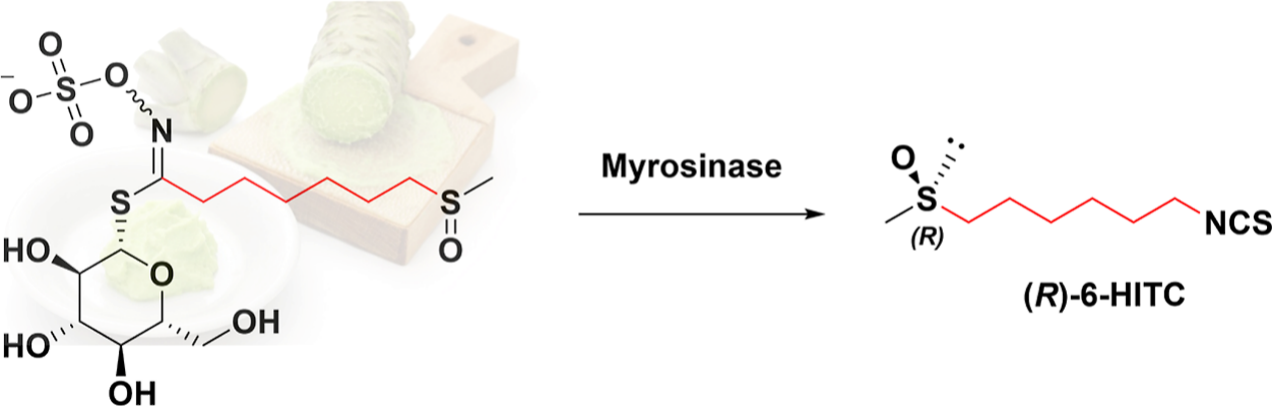
Biological Synthesis of (*R*)-6-HITC from Wasabi GLS

The structural features of natural ITCs appear to play a key role in their biological activities. Small changes in their structure such as the length of the alkyl chain between the two functional groups, sulfoxide and ITC, have been shown to have a significant impact on their chemopreventive effects.^17−19^ In nature, in addition to the common glycolic moiety, ITC molecules possess a variable amino acid-derived side chain,^20^ which in the case of 6-HITC is characterized by the presence of a sulfinyl group (Scheme 1).^21,22^ Previous in vitro studies have demonstrated significant pharmacological effects of 6-HITC, including anti-inflammatory effects in lipopolysaccharide (LPS)-activated RAW264 murine macrophage cells^23,24^ and in a murine model of acute and chronic dextran sulfate sodium (DSS)-induced colitis.^16^ Notable antiplatelet effects have also been observed in vitro and in rats,^25^ as well as chemopreventive effects in melanoma and human breast cancer cell lines.^26^ However, these studies have provided limited information about the molecular mechanisms involved in the pharmacological effects.

On the other hand, it is reported that sulfur chirality could have significant biological activity. This fact has been confirmed for other ITCs such as sulforaphane (SFN).^27^ In this sense, natural 6-HITC exists as a unique enantiomer; however, so far, preclinical studies have been carried out using its racemic form. Therefore, the importance of sulfur chirality in the biological activity of 6-HITC is unknown. Consequently, we consider it of interest to develop a method for the selective synthesis of natural enantiomer *R*.

In the present work, in order to obtain enantiomerically pure (*R*)-6-HITC, we have applied the “DAG-methodology”. This method was developed within our research group and stands out as the preferred methodology for numerous reasons. It allows for the synthesis of a diastereomerically pure sulfinate ester, featuring an alkyl chain on the sulfinyl sulfur, as a precursor to the desired sulfoxide upon treatment with a nucleophile. Moreover, the appropriate choice of the nature of the base present in the medium facilitates the accessibility of both epimers on sulfur in an enantiodivergent manner. This is achieved through a dynamic kinetic resolution of the initial sulfinyl chloride, showcasing the versatility and efficiency of the process.^28,29^

Given this background, based on these premises, and as part of our research program on SFN analogues, we decided to study the effect of the chain linking the electrophilic ITC group and the Lewis basic sulfinyl moiety on the (*R*)-6-HITC anti-inflammatory effect. Thus, this study was carried out to evaluate the immunomodulatory activity and regulatory mechanisms that underpin the potentially beneficial effects of the natural enantiomer (*R*)-6-HITC in an *ex vivo* model of inflammation, using murine peritoneal macrophage stimulated by LPS, to initially validate its future use as a nutraceutical compound.

## 2 Materials and Methods

### 2.1 Chemicals and Instruments

For the reactions run under an atmosphere of dry argon, oven-dried glassware and dried solvents were used. Chemicals were obtained from commercial sources and were used without further purification. TLC was carried out on silica gel GF254 (Merck), and compounds were detected by charring with phosphomolybdic acid/EtOH. For flash chromatography, a Merck 230–400 mesh silica gel was used. Chromatographic columns were eluted with a positive pressure of air, and eluents were given as volume-to-volume ratios (v/v). Nuclear magnetic resonance (NMR) spectra were recorded with Bruker Avance 500 MHz spectrometers. Chemical shifts are reported in parts per million, and coupling constants are reported in Hz. High-resolution mass spectra (HRMS) were recorded in the Centro de Investigación, Tecnología e Innovación in the University of Seville, with Kratos MS-80RFA 241-MC equipment. Optical rotations were determined with a PerkinElmer 341 polarimeter. Enantiomeric excesses were measured with a Waters Alliance 2695 and Agilent Technologies 1200 series high-performance liquid chromatography (HPLC) apparatus with stationary chiral phase columns (Chiralcel). The synthetic route (ii–viii steps) was performed according to the methodology described by Posner et al.^18^

#### 2.1.1 Synthesis of 6-Azidohexan-1-ol (**1**)

To a solution of 6-chloro-1-hexanol (97%) (30.9 g, 219.59 mmol) in dry DMF (180 mL) under an argon atmosphere was added sodium azide (28.6 g, 439.17 mmol). The reaction mixture was heated to 50 °C overnight. After completion of the reaction, water (150 mL) was added and extracted with CH_2_Cl_2_ twice. The resulting organic layers were dried over anhydrous Na_2_SO_4_, and the solvent was evaporated to give 31.4 g (219.31 mmol, quantitative yield) of 1 as a colorless oil, which was used without further purification. *R*_f_ = 0.35 (CH_2_Cl_2_); ^1^H NMR (500 MHz, CDCl_3_), 3.60 (t, 2H, *J* = 6.5 Hz), 3.23 (t, 2H, *J* = 6.9 Hz), 1.60–1.51 (m, 5H), and 1.38–1.34 (m, 4H) ppm; ^13^C NMR (125 MHz, CDCl_3_): δ 62.9, 51.6, 32.7, 29.0, 26.7, and 25.5 ppm; HRMS (FAB) calcd. for C_6_H_13_N_3_O (M)^+^*m*/*z* 143.1059, found *m*/*z* 143.1059. Supporting Information contains ^1^H and ^13^C NMR (Figures S1 and S2, respectively).

#### 2.1.2 Synthesis of 6-Azidohexyl Methanesulfonate (**2**)

To a solution of 6-azidohexan-1-ol 1 (31.4 g, 219.31 mmol) and Et_3_N (40.0 mL, 285.10 mmol) in dry THF (170 mL), under an argon atmosphere and at 0 °C, methanesulfonyl chloride (22.1 mL, 285.10 mmol) was added dropwise. After 2 h (h) of stirring at room temperature, the reaction mixture was quenched with saturated NH_4_Cl aqueous solution and extracted with CH_2_Cl_2_ twice. Then the combined organic layers were washed with a saturated NaCl aqueous solution and dried over anhydrous Na_2_SO_4_. The solvent was evaporated to give 47.2 g (213.21 mmol, 97% yield) of 2 as a colorless oil, which was used without further purification. *R*_f_ = 0.25 (CH_2_Cl_2_/hexane, 4:1); ^1^H NMR (500 MHz, CDCl_3_): δ 4.21 (t, 2H, *J* = 6.5 Hz), 3.26 (t, 2H, *J* = 6.8 Hz), 3.00 (s, 3H), 1.78–1.72 (m, 2H), 1.63–1.57 (m, 2H), and 1.46–1.38 (m, 4H) ppm; ^13^C NMR (125 MHz, CDCl_3_): δ 70.0, 51.4, 37.5, 29.2, 28.8, 26.3, and 25.2 ppm; HRMS (FAB) calcd. for C_7_H_15_N_3_O_3_NaS (M + Na)^+^: *m*/*z* 244.0732, found *m*/*z* 244.0737. Supporting Information contains ^1^H and ^13^C NMR (Figures S3 and S4, respectively).

#### 2.1.3 Synthesis of 6-Azidohexyl-1-thioacetate (**3**)

To a solution of 6-azidohexyl methanesulfonate 2 (35.0 g, 158.32 mmol) in dry DMF (500 mL) and under an argon atmosphere, potassium thioacetate (23.5 g, 205.85 mmol) was added at room temperature. The reaction mixture was stirred overnight, washed with water, and extracted several times with EtOAc. The combined organic phases were washed with saturated NaHCO_3_ aqueous solution and brine, dried over anhydrous Na_2_SO_4_, and evaporated to obtain 30.7 g (152.36 mmol, 96% yield) of 3 as a brown oil, which was used without further purification. *R*_f_ = 0.30 (hexane/EtOAc, 95:5); ^1^H NMR (500 MHz, CDCl_3_): δ 3.24 (t, 2H, *J* = 7.0 Hz), 2.85 (t, 2H, *J* = 7.3 Hz), 2.31 (s, 3H), 1.60–1.54 (m, 4H), and 1.39–1.36 (m, 4H) ppm; ^13^C NMR (125 MHz, CDCl_3_): δ 196.1, 51.5, 30.8, 29.6, 29.1, 28.9, 28.4, and 26.4 ppm; HRMS (FAB) calcd. for C_8_H_15_N_3_ONaS (M + Na)^+^: *m*/*z* 224.0834, found *m*/*z* 224.0831. Supporting Information contains the ^1^H and ^13^C NMR (Figures S5 and S6, respectively).

#### 2.1.4 Synthesis of 6-Azidohexane-1-sulfinyl Chloride (**4**)

To a solution of thioacetate 3 (15.5 g, 76.85 mmol) in methylene chloride (67 mL) at −20 °C were added acetic anhydride (7.3 mL, 76.85 mmol) and sulfuryl chloride (12.4 mL, 153.71 mmol). The resulting mixture was stirred for 1 h at −5 °C and then the solvent was evaporated, and the residue was dried under vacuum to give 16.1 g (76.80 mmol, quantitative yield) of 4 as a black low-melting-point solid. The crude sulfinyl chloride, which was kept under argon, was used without further purification in the following reaction for the preparation of sulfinate esters. ^1^H NMR (300 MHz, CDCl_3_): δ 3.38 (t, 2H, *J* = 7.7 Hz), 3.28 (t, 2H, *J* = 6.7 Hz), 1.99–1.89 (m, 2H), 1.67–1.58 (m, 2H), and 1.55–1.39 (m, 4H) ppm. Supporting Information contains ^1^H NMR (Figures S7).

#### 2.1.5 Synthesis of (*S*)-(1,2:5,6-Di-*O*-isopropylidene-α-d-glucofuranosyl) 6-Azidohexanesulfinate (**5-(*S*)**)

To a solution of 1,2:5,6-di-*O*-isopropylidene-α-d-glucofuranosyl (DAGOH) (7.3 g, 28.00 mmol) and DIPEA (9.8 mL, 56.00 mmol) in anhydrous toluene (200 mL), cooled to −78 °C and placed under an argon atmosphere, 6-azidohexane-1-sulfinyl chloride 4 (7.1 g, 33.70 mmol) was added while the reaction mixture was being vigorously stirred. After stirring at −78 °C for 1 h, the reaction mixture was treated with 1 M HCl aqueous solution and extracted with CH_2_Cl_2_. The combined organic layers were successively washed with saturated NaHCO_3_ aqueous solution and brine, dried over Na_2_SO_4_, and evaporated to obtain the S sulfinate as the major diastereomer with 84% diastereomeric excess. The crude was purified by column chromatography (hexane/2-propanol 20:1) to give 9.5 g (21.96 mmol, 78% yield) of diastereomerically pure **5-(*S*)** as a yellow oil. *R*_f_ = 0.26 (hexane/2-propanol, 10:1); [α]_D_ = −39.3 (*c* = 1.0, CHCl_3_); ^1^H NMR (300 MHz, CDCl_3_) δ 5.90 (d, 1H, *J* = 3.6 Hz), 4.74 (d, 1H, *J* = 2.5 Hz), 4.60 (d, 1H, *J* = 3.70 Hz), 4.32–4.24 (m, 2H), 4.09 (dd, 1H, *J* = 8.5 and 5.8 Hz), 4.01 (dd, 1H, *J* = 8.5 and 5.0 Hz), 3.27 (t, 2H, *J* = 6.74 Hz), 2.88–2.70 (m, 2H), 1.78–1.67 (m, 2H), 1.66–1.56 (m, 2H), 1.51 (s, 3H), 1.44–1.36 (m, 4H), 1.43 (s, 3H), 1.34 (s, 3H), and 1.31 (s, 3H) ppm; ^13^C NMR (75 MHz, CDCl_3_): δ 112.4, 109.2, 104.9, 83.6, 80.3, 79.2, 72.3, 66.7, 57.1, 51.2, 28.5, 28.3, 26.7, 26.7, 26.3, 26.2, 25.2, and 21.1 ppm; HRMS (FAB) calcd. for C_18_H_31_N_3_O_7_NaS (M + Na)^+^: *m*/*z* 456.1780, found *m*/*z* 456.1780. Supporting Information contains the ^1^H and ^13^C NMR (Figures S8 and S9, respectively).

#### 2.1.6 Synthesis of (*R*)-(−)-1-Azido-6-(methylsulfinyl)-hexane (**6-(*R*)**)

To a solution of sulfinate **5-(*S*)** (2.9 g, 6.66 mmol) in anhydrous toluene (20 mL), at 0 °C, was added methyl magnesium bromide 1.4 M (7.2 mL, 10.00 mmol). After stirring for 1 h at 0 °C, saturated NH_4_Cl aqueous solution was added. The aqueous layer was extracted with CH_2_Cl_2_, and the resulting organic layers were combined, dried on Na_2_SO_4_, and concentrated. The crude product was purified by column chromatography (EtOAc/MeOH 15:1) to give 926 mg of **6-(*R*)** (4.86 mmol, 73% yield) as a colorless liquid. *R*_f_ = 0.17 (EtOAc/MeOH, 9:1); [α]_D_ = −64.24 (*c* = 1.1, CHCl_3_); ^1^H NMR (500 MHz, CDCl_3_): δ 3.26 (t, 2H, *J* = 6.9 Hz), 2.77–2.58 (m, 2H), 2.55 (s, 3H), 1.82–1.72 (m, 2H), and 1.65–1.36 (m, 6H); ^13^C NMR (125 MHz, CDCl_3_): δ 54.7, 51.4, 38.8, 28.7, 28.5, 26.5, and 22.6; HRMS (FAB) *m*/*z* calcd. for C_7_H_16_N_3_OS (M + H)^+^: 190.1014, found: 190.1021. Supporting Information contains the ^1^H and ^13^C NMR (Figures S10 and S11, respectively).

#### 2.1.7 Synthesis of (*R*)-(−)-1-Isothiocyanato-6-(methylsulfinyl)-hexane (**(*R*)-6-HITC**)

To a solution of azide **6-(*R*)** (816 mg, 4.32 mmol) in Et_2_O (3 mL) was added triphenylphosphine (2.2 g, 8.20 mmol), and the reaction was refluxed for 1 h. After removing the solvent in a vacuum, carbon disulfide (7 mL) was added, and the mixture was refluxed for 3 h. Finally, the solvent was removed under vacuum, and the crude product was purified by column chromatography (EtOAc/MeOH 9:1) to give 719 mg of **(*R*)-6-HITC** (3.50 mmol, 81% yield) as a colorless liquid. *R*_f_ = 0.4 (EtOAc/MeOH, 9:1); [α]_D_ = −70.62 (*c* = 0.5, CHCl_3_); ^1^H NMR (500 MHz, CDCl_3_): δ 3.51 (t, 2H, *J* = 10.5 Hz), 2.74–2.61 (m, 2H), 2.55 (s, 3H), 1.83–1.68 (m, 2H), 1.54–1.44 (m, 2H), and 1.54–1.48 (m, 4H) ppm; ^13^C NMR (125 MHz, CDCl_3_): δ 130.4, 54.5, 45.0, 38.8, 29.7, 28.0, 26.3, and 22.5 ppm; HRMS *m*/*z* calcd. for C_8_H_16_NOS_2_ (M + H)^+^: 206.0673, found: 206.0669; HPLC: ASH Chiracel column, (*n*-hexane/isopropanol 40:60; 1.0 mL/min; 23 °C) *t*_R_ = 16.4 min (*R*-isomer), *t*_R_ = 19.5 min (*S*-isomer). Supporting Information contains the ^1^H and ^13^C NMR (Figures S12 and S13, respectively) and HPLC chromatogram (Figure S14).

### 2.2 Experimental Animals

Female Swiss mice (25–30 g) from the Animal Production Centre of the University of Seville were used and were allowed free access to food and water throughout the study. The experiments were performed at the Department of Pharmacology (University of Seville, Spain). The procedures for handling and care of the animals were approved by the Ethical Committee of the University of Seville (CEEA-US2022–18) and by the Consejería de Agricultura, Pesca y Desarrollo (Junta de Andalucía, 12/04/2023/010), according to RD 53/1 February 2013, under European Union guidelines (EU Directive 2020/569).

### 2.3 Macrophage Extraction and Culture

Mice were injected intraperitoneally with 1 mL of 3.8% w/v sodium thioglycolate (BD Difco, Le Pont de Claix, France). Once the animals were sacrificed 72 h later by CO_2_ exposure, the intraperitoneal cavity was washed with cold sterile phosphate buffer saline solution (PBS) to remove the cellular exudate. The extracted cells were centrifuged and resuspended in RPMI 1640 culture medium with l-glutamine enriched with 10% heat-inactivated fetal calf serum (PAA, Pasching, Austria) and antibiotics (100 mg/mL streptomycin and 100 U/ml penicillin). Nonadherent cells were removed with PBS, and the medium was replaced with RPMI 1640 containing the treatment (6.25 or 12.5 μM of (*R*)-6-HITC) or the vehicle, dimethyl sulfoxide (DMSO) (Sigma-Aldrich, St. Louis, MO, USA), after 2 h of incubation. Subsequently, macrophages were stimulated with LPS from *Escherichia coli* (Sigma-Aldrich, St. Louis, MO, USA) for 18 h after 30 min. Lastly, both cell pellet and supernatant were stored at −80 °C for Western blot and ELISA assays, respectively.

### 2.4 Determination of Cellular Viability

Macrophages were cultured in 96-well plates at a density of 1 × 10^5^ cells/well and incubated in the presence or absence of a wide range of (*R*)-6-HITC concentrations (200 μM-1.6 μM) for 18 h. Subsequently, the cells were fixed by adding 50 μL of a 50% w/v solution of cold trichloroacetic acid (Sigma-Aldrich, St. Louis, MO, USA) and incubated for 1 h at 4 °C. After that, the plates were washed five times with deionized water. Then, 100 μL of a 0.4% w/v solution of sulforhodamine B (SRB) (Sigma-Aldrich, St. Louis, MO, USA) was added to each well, and the mixture was incubated for 30 min at room temperature. Then, the plates were washed with 1% (v/v) acetic acid solution (Panreac, Barcelona, Spain). Finally, 100 μL of a Tris base solution (pH 10.5; 10 mmol/L) (Sigma-Aldrich, St. Louis, MO, USA) was added.

Cell viability was determined by measuring the optical density at 510 nm using a multiwell plate reader spectrophotometer ELx800 (BioTek, Bad Friedrichshall, Germany). The cell survival was calculated as a percentage of absorbance by comparing the treated cells with untreated control cells (representing 100% cell survival).

The viability in each experiment was always equal to or greater than 80%. The (*R*)-6-HITC stock solution was prepared in DMSO (Sigma-Aldrich, St. Louis, MO, USA), and the solution was diluted to the desired concentrations in the culture medium. The concentration of DMSO in the culture medium was less than 1% (v/v) in all experiments and did not exert any significant effects on the cells.

### 2.5 Quantification of Nitrite Levels by Griess Assay

Several techniques have been developed for the indirect determination of this gaseous molecule due to the short half-life of nitric oxide (NO). One of them is the Griess assay, by which the nitrite ion levels present in the sample are quantified spectrophotometrically.^30^ A standard curve of sodium nitrite and 100 μL of cellular supernatants were transferred to a 96-well plate and subsequently mixed with the Griess reagent (Sigma-Aldrich, St. Louis, MO, USA). The mixture was then incubated at room temperature for 15 min. Finally, the absorbance at 540 nm was measured using an ELISA reader (BioTek, Bad Friedrichshall, Germany). The quantification of the nitrite content was determined as the NO generation index, extrapolating from a standard curve established with sodium nitrite. The results were expressed as a percentage of nitrite production in relation to the DMSO-LPS treated cells (stimulated cells that were not subjected to any treatment).

### 2.6 Detection of Intracellular Reactive Oxygen Species

The determination of intracellular reactive oxygen species (ROS) levels was conducted utilizing the 2′,7′-dichlorofluorescin-diacetate (DCFDA) assay kit (Abcam, Cambridge, UK) following the manufacturer’s guidelines. A total of 2.5 × 10^4^ cells/well were seeded onto a black 96-well plate that had been pretreated with (*R*)-6-HITC (12.5 and 6.25 μM). After 30 min, both treated and untreated cells were stimulated with LPS. Subsequently, DCFDA (25 μM) was added to each well, and the cells were incubated at 37 °C for 45 min. A fluorescence microplate reader (SynergyTM HTX Biotek, Bad Friedrichshall, Germany) was employed to measure the excitation and emission wavelengths at 485 and 535 nm, respectively. H_2_O_2_ (Sigma-Aldrich, Cambridge, UK) was used as the positive pro-oxidant control, representing 100% intracellular ROS production.

### 2.7 Quantification Levels of Pro-inflammatory Cytokines IL-1β, IL-6, IL-17, and TNF-α

The cell-free supernatants derived from the culture of mouse peritoneal macrophages were harvested 18 h after LPS stimulation. Subsequently, these supernatants were analyzed using enzyme-linked immunoassay kits (ELISA) to quantify the concentrations of interleukin (IL)-1β (BD OptEIA, San Jose, CA, USA), IL-6 (Diaclone, Besacon Cedex, France), IL-17, and tumor necrosis factor (TNF)-α (Peprotech, London, UK), following the instructions provided by the respective manufacturers, by spectrophotometry using the iMARK plate reader (Bio-Rad, Hercules, CA, USA).

### 2.8 Immunoblotting Assay

Protein concentrations in the samples were determined using the protein assay reagent (Bio-Rad, Hercules, CA, USA) with γ-globulin as a standard, following the procedure outlined by Bradford.^31^ Western blot assay was performed following the methodology described by Alcarranza et al.^32^ Nitrocellulose membranes were incubated overnight at 4 °C with specific primary antibodies: rabbit anti-iNOS, rabbit anti-pSTAT3, rabbit anti-pJNK, rabbit anti-pp38, rabbit anti-pERK 1/2, rabbit anti-NLRP3, rabbit anticaspase-1, rabbit anti-COX-2, rabbit anti-pJAK2, rabbit anti-JNK, rabbit anti-p38, mouse anti-ERK 1/2 (Cell Signaling Technology, Danvers, MA, USA) (1:1000), rabbit anti-IL18, rabbit anti-mPGES1 (Abcam, Cambridge, UK) (1:1000), rabbit anti-HO-1 (Enzo, Madrid, Spain) (1:1000), and rabbit anticaspase-11 (Novus Biologicals, Littleton, CO, USA) (1:500). After washing, the membranes were incubated with horseradish-peroxidase-labeled secondary antibody, either antirabbit (Cell Signaling Technology, Danvers, MA, USA) (1:2000) or antimouse (Dako, Atlanta, GA, USA) (1:2000), in a blocking solution for 1–2 h at room temperature. To confirm equal loading, the blots were probed for β-actin expression using a mouse anti-β-actin antibody (Abcam, Cambridge, UK) (1:10,000). Immunodetection was carried out using an enhanced chemiluminescence light detection kit (Pierce, Rockford, IL, USA).

The immune signals were captured using the Amersham Imager 600 from GE Healthcare (Buckinghamshire, UK), and the densitometric data were analyzed after normalization to the housekeeping loading control. The signals were quantified and analyzed using Image Processing and Analysis FijiImageJ software (W. Rasband, National Institutes of Health) and expressed relative to the DMSO-LPS treated cells.

### 2.9 Statistical Analysis

Data were evaluated with Graph Pad Prism version 5.01 software (San Diego, CA, USA). All values in the figures and text are expressed as arithmetic means ± standard error of the mean (SEM). One-way analysis of variance was used to evaluate the statistical significance of any difference in each parameter between groups, and after that, Tukey’s multiple comparison test was used as a post hoc test. *p* values <0.05 were considered statistically significant.

## 3 Results

### 3.1 Impact of (*R*)-6-HITC on the Survival of Murine Peritoneal Macrophages

**Figure 1:**
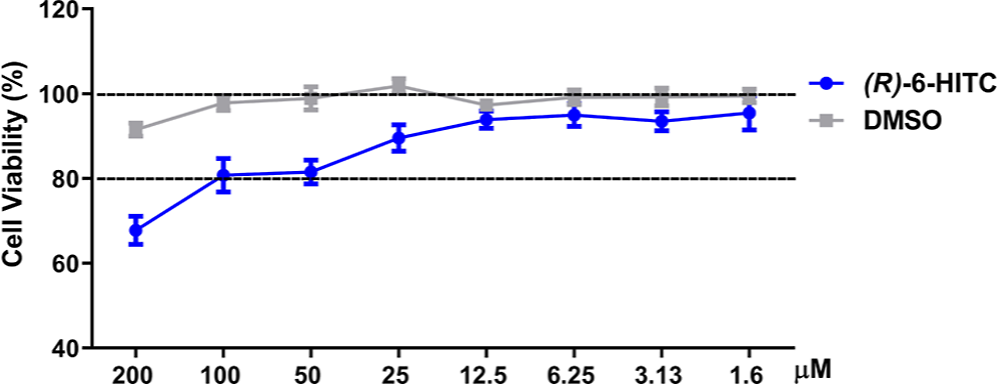
Effect of (*R*)-6-HITC on cell survival. The survival rate was expressed as the percentage of viability with respect to 100% of control untreated cells. Results are presented as mean ± SEM of at least six independent experiments.

In order to investigate whether this natural ITC influences the macrophage cell viability, an SRB assay was performed. Several concentrations of this compound (200–1.6 μM) were used to treat immune cells for 18 h. (*R*)-6-HITC was not harmful to cell survival (≥80%) at concentrations 1.6–100 μM. DMSO was used as a vehicle, and it did not affect cell viability (Figure 1).

### 3.2 (*R*)-6-HITC Ameliorates the Production of IL-1β, IL-6, IL17, and TNF-α

**Figure 2:**
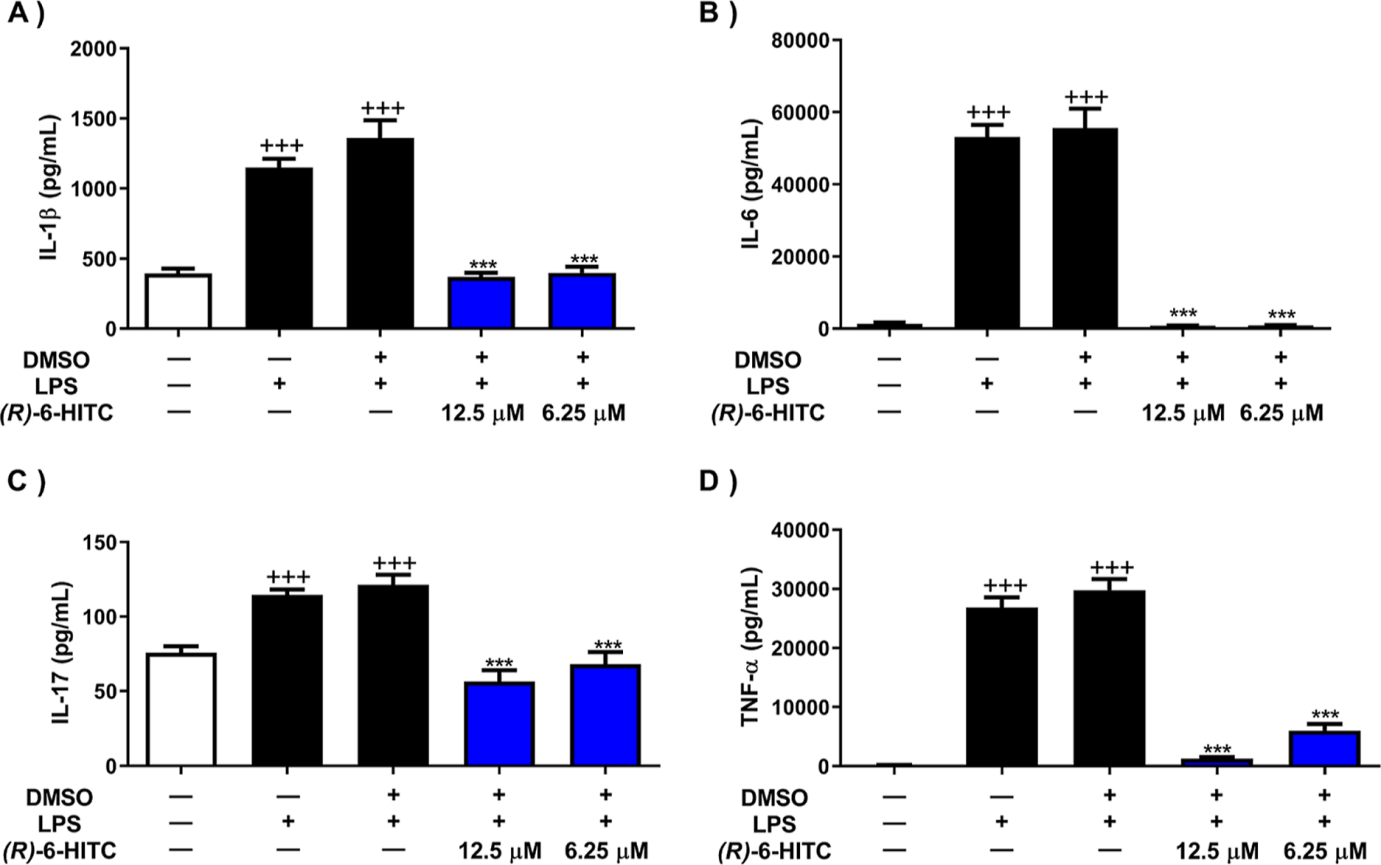
Effects of (*R*)-6-HITC on IL-1β (A), IL-6 (B), IL-17 (C), and TNF-α (D) pro-inflammatory cytokines. Macrophages were pretreated with the indicated concentrations of (*R*)-6-HITC for 30 min followed by stimulation with 5 μg/mL LPS for 18 h. The level of cytokines was measured by ELISA in cell supernatants. Data are expressed as mean ± SEM (*n* = 8). (+++) *p* < 0.001 vs control cells (unstimulated); (***) *p* < 0.001 vs LPS-DMSO-treated cells.

We explored whether (*R*)-6-HITC might influence the secretion of pro-inflammatory cytokines IL-1β, IL-6, IL-17, and TNF-α in LPS-stimulated murine peritoneal macrophages. As can be seen in Figure 2, LPS-stimulated cells experienced a significant increase in the production of these cytokines (+++*p* < 0.001 vs unstimulated cells). Nevertheless, treatment with 12.5 and 6.25 μM of (*R*)-6-HITC significantly ameliorated the overproduction of these pro-inflammatory markers compared to the LPS-DMSO group (***p < 0.001 vs LPS-DMSO stimulated cells).

### 3.3 LPS-Induced COX-2 and mPGES-1 Enzyme Expression was Reduced by (*R*)-6-HITC

**Figure 3:**
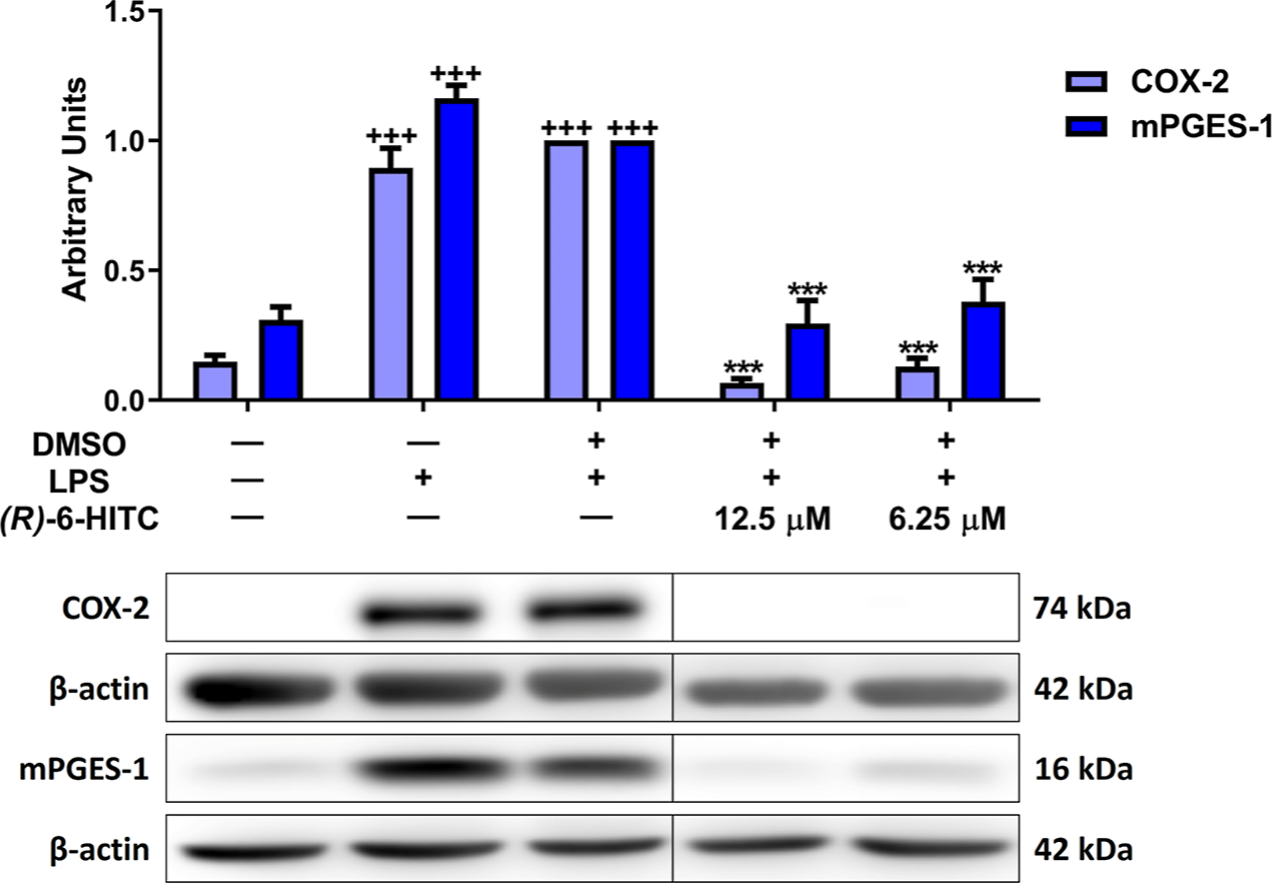
(*R*)-6-HITC decreased COX-2 and mPGES-1 protein expression in peritoneal macrophages stimulated by LPS. Peritoneal murine cells were treated with (*R*)-6-HITC (12.5 and 6.25 μM). Thirty min later, cells were exposed to LPS (5 μg/mL) for 18 h. β-actin housekeeping gene was used to normalize the densitometric analysis of COX-2 or mPGES-1 protein. Data are represented as means ± SEM (*n* = 6). (+++) *p* < 0.001 vs unstimulated control cells; (***) *p* < 0.001 vs LPS-DMSO-treated cells.

To assess the anti-inflammatory activity of (*R*)-6-HITC on macrophages, cyclooxygenase (COX)-2 and microsomal prostaglandin E synthase-1 (mPGES-1) protein expression was analyzed by Western blotting. From Figure 3, a significant overexpression of COX-2 and mPGES-1 proteins in LPS-stimulated cells (+++*p* < 0.001 vs nonstimulated control cells) can be noted. Nonetheless, (*R*)-6-HITC pretreatment (12.5 and 6.25 μM) significantly reduced both proteins’ expression in comparison to that in LPS-DMSO cells (***p < 0.001 vs LPS-DMSO-treated cells).

### 3.4 (*R*)-6-HITC Decreased Intracellular ROS Levels and Nitrite Production and Downregulated iNOS Protein Expression in Murine Peritoneal Macrophages Stimulated by LPS

**Figure 4:**
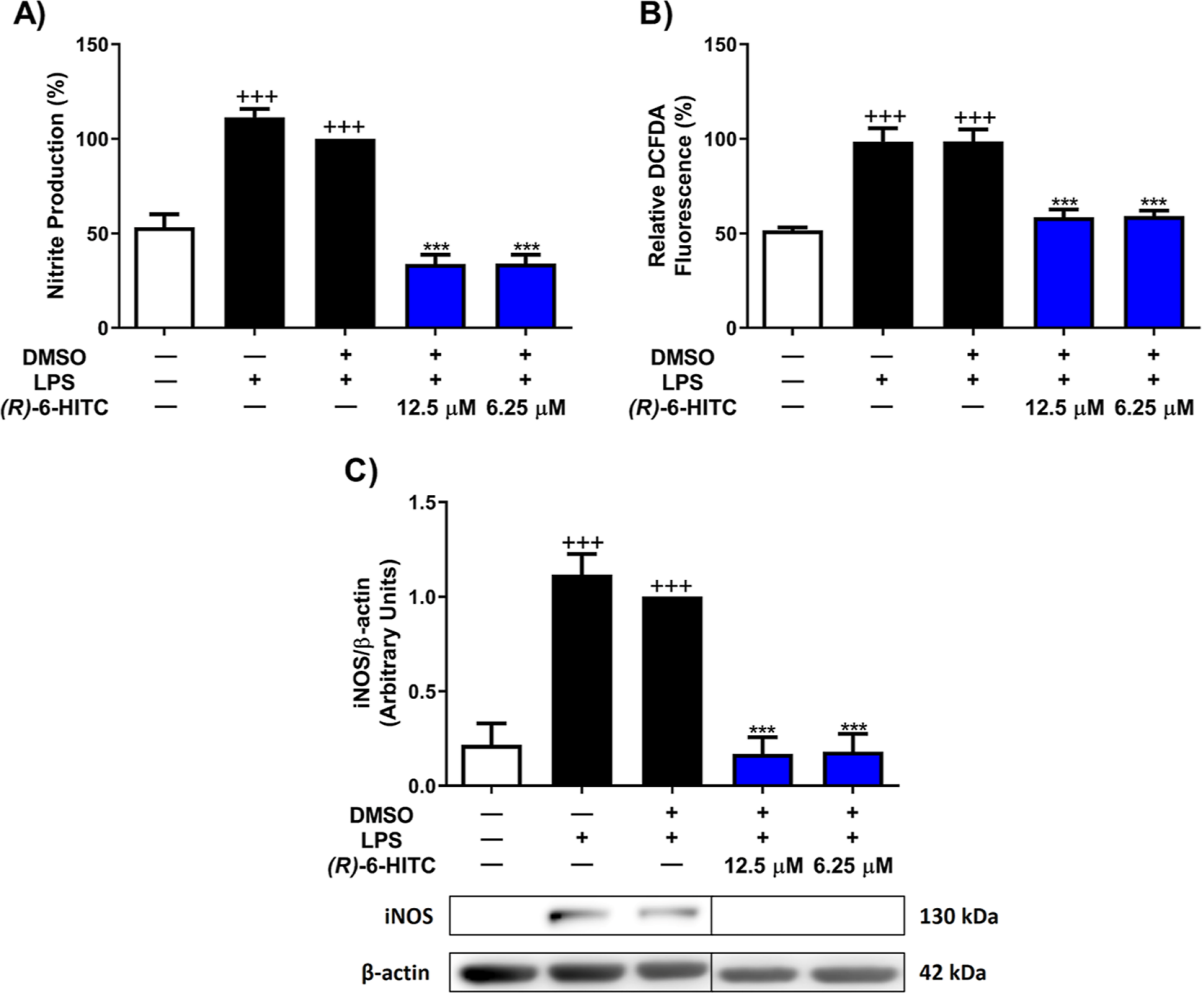
(*R*)-6-HITC decreases the production of nitrites (A), ROS levels (B), and iNOS protein expression (C). Analyses of NO and ROS levels were determined in the cell supernatant while the protein expression of iNOS was carried out in the cell lysate. (*R*)-6-HITC (12.5 and 6.25 μM) was used to treat immune cells for 30 min. Later, cells were exposed to LPS for 18 h. The normalization of the densitometry was performed by measuring to β-actin housekeeping gene. Data shown are means ± SEM (*n* = 6). (+++) *p* < 0.001 vs unstimulated control cells; (***) *p* < 0.001 vs LPS-DMSO-treated cells.

To assess the role of (*R*)-6-HITC on LPS-induced oxidative stress in murine peritoneal macrophages, nitrite levels, ROS, and inducible nitric oxide synthase (iNOS) protein expression were measured by Griess, DCFDA, and Western blot assays, respectively. As it can be seen in Figure 4, LPS stimulation remarkably increased the production of ROS, nitrites, and iNOS expression (+++*p* < 0.001 vs unstimulated control cells). However, pretreatment with (*R*)-6-HITC was able to significantly reduce ROS levels, iNOS protein expression, and subsequently nitrite levels, compared to those in LPS-DMSO stimulated cells, revealing a potent antioxidant effect (***p < 0.001 vs LPS-DMSO cells).

### 3.5 (*R*)-6-HITC Upregulated Nrf2/HO-1 Axis Protein Expression in LPS-Stimulated Murine Peritoneal Macrophages

**Figure 5:**
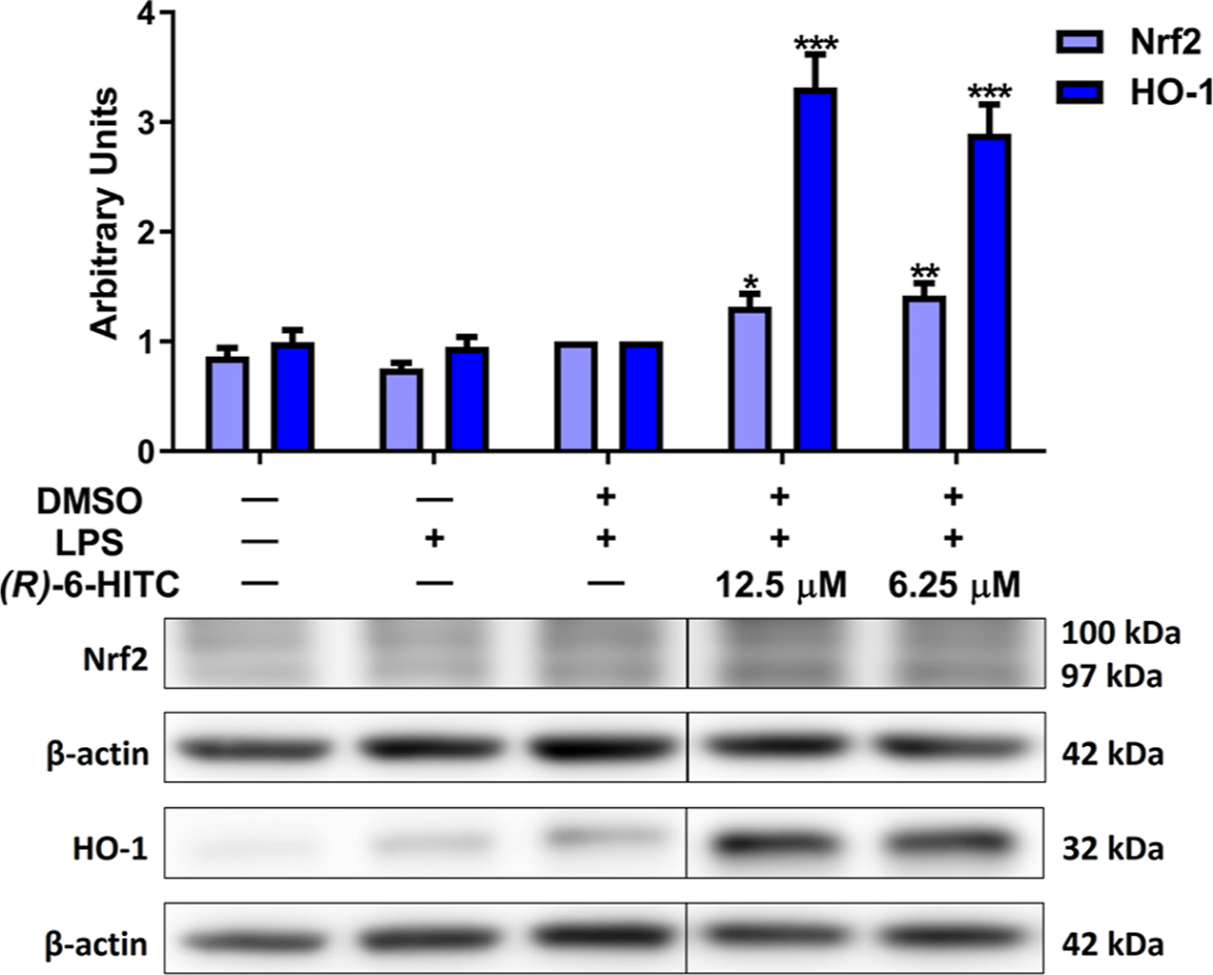
Effects of (*R*)-6-HITC on the expression of Nrf2 and HO-1 in LPS-activated immune cells. Murine macrophages were pretreated with (*R*)-6-HITC (12.5 and 6.25 μM) for 30 min, followed by LPS stimulation for 18 h. To normalize densitometric analysis of Nrf2 and HO-1, β-actin housekeeping gene was used. Data shown are means ± SEM (*n* = 6). (*) *p* < 0.05; (**) *p* < 0.01; (***) *p* < 0.001 vs LPS-DMSO-treated cells.

Figure 5 illustrates the impact of (*R*)-6-HITC on the nuclear factor erythroid 2-related factor 2/heme oxygenase-1 (Nrf2/HO-1) signaling pathway. Compared with the LPS-DMSO control cells, both concentrations of (*R*)-6-HITC significantly elevated Nrf2 (*p < 0.05 and **p < 0.01) and HO-1 (***p < 0.001) protein expression. Considering these results together with the decrease in ROS and NO production and iNOS protein expression, we could confirm the potent antioxidant effects of (*R*)-6-HITC.

### 3.6 Effects of (*R*)-6-HITC on MAPKs Activation in LPS-Activated Peritoneal Macrophages

**Figure 6:**
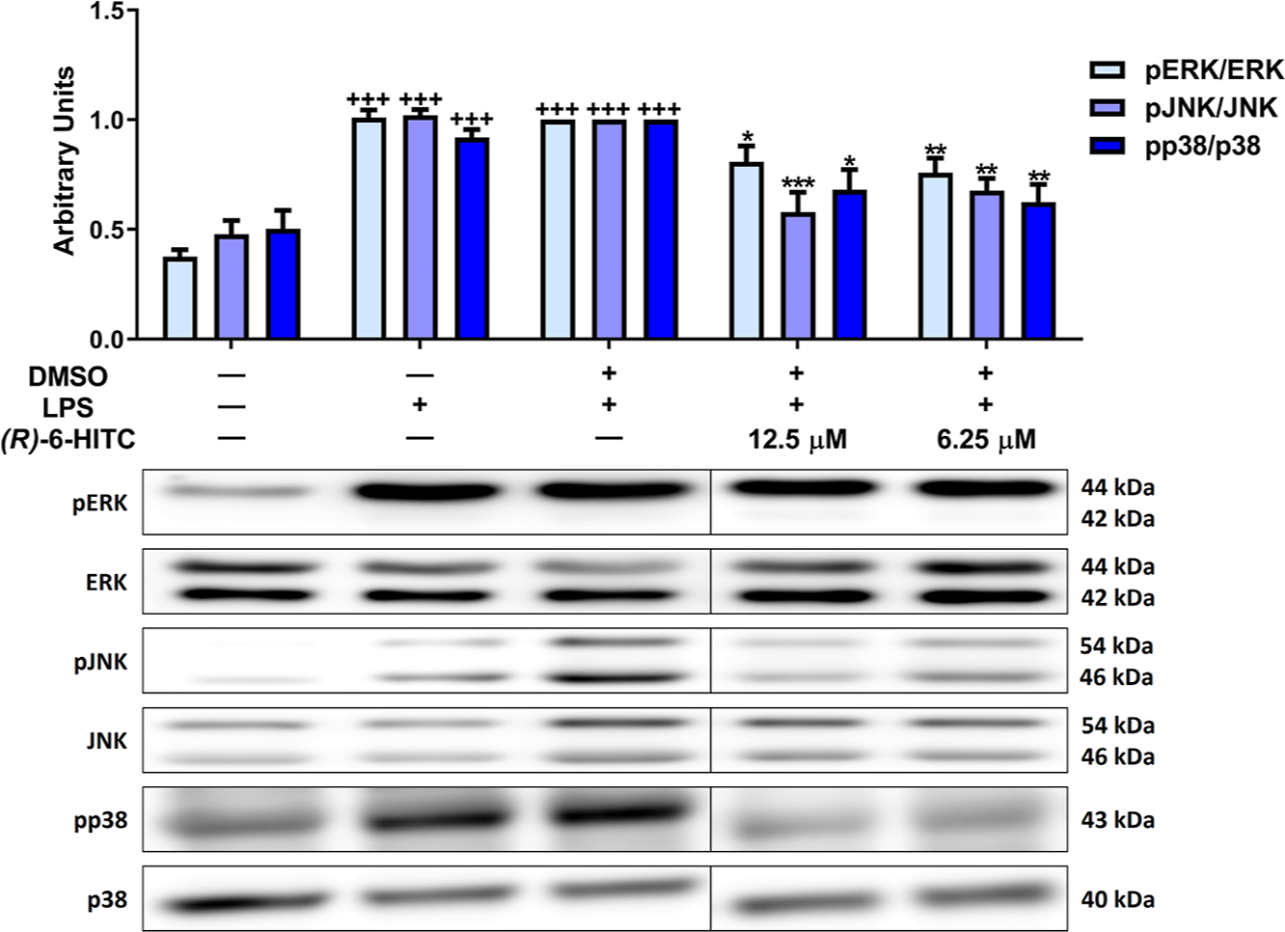
Effects of (*R*)-6-HITC in the phosphorylation of ERK, p38, and JNK on LPS-activated immune cells. Macrophages were pretreated with both concentrations of (*R*)-6-HITC (12.5 and 6.25 μM) for 30 min, followed by LPS stimulation for 18 h. To normalize densitometric analysis of pERK, pp38, and pJNK, ERK, p38, and JNK housekeeping genes were used, respectively. Data shown are means ± SEM (*n* = 6). (+++) *p* < 0.001 vs control cells unstimulated; (*) *p* < 0.05; (**) *p* < 0.01; (***) *p* < 0.001 vs LPS-DMSO-treated cells.

To delve into the molecular mechanisms responsible for the effects of (*R*)-6-HITC, the activation of mitogen-activated protein kinases (MAPKs) was evaluated by Western blot. Figure 6 shows a significant increase in the level of phosphorylation of p38, extracellular signal-regulated kinase (ERK)1/2, and c-Jun N-terminal kinase (JNK) upon stimulating macrophages with LPS (+++*p* < 0.001 vs unstimulated control cells). Nevertheless, both concentrations of (*R*)-6-HITC were able to significantly decrease ERK, p38, and JNK phosphorylation (*p < 0.05, **p < 0.01; ***p < 0.001 vs LPS-DMSO-treated cells).

### 3.7 (*R*)-6-HITC Inhibited JAK/STAT Signaling Pathway in LPS-Induced Murine Peritoneal Macrophages

**Figure 7:**
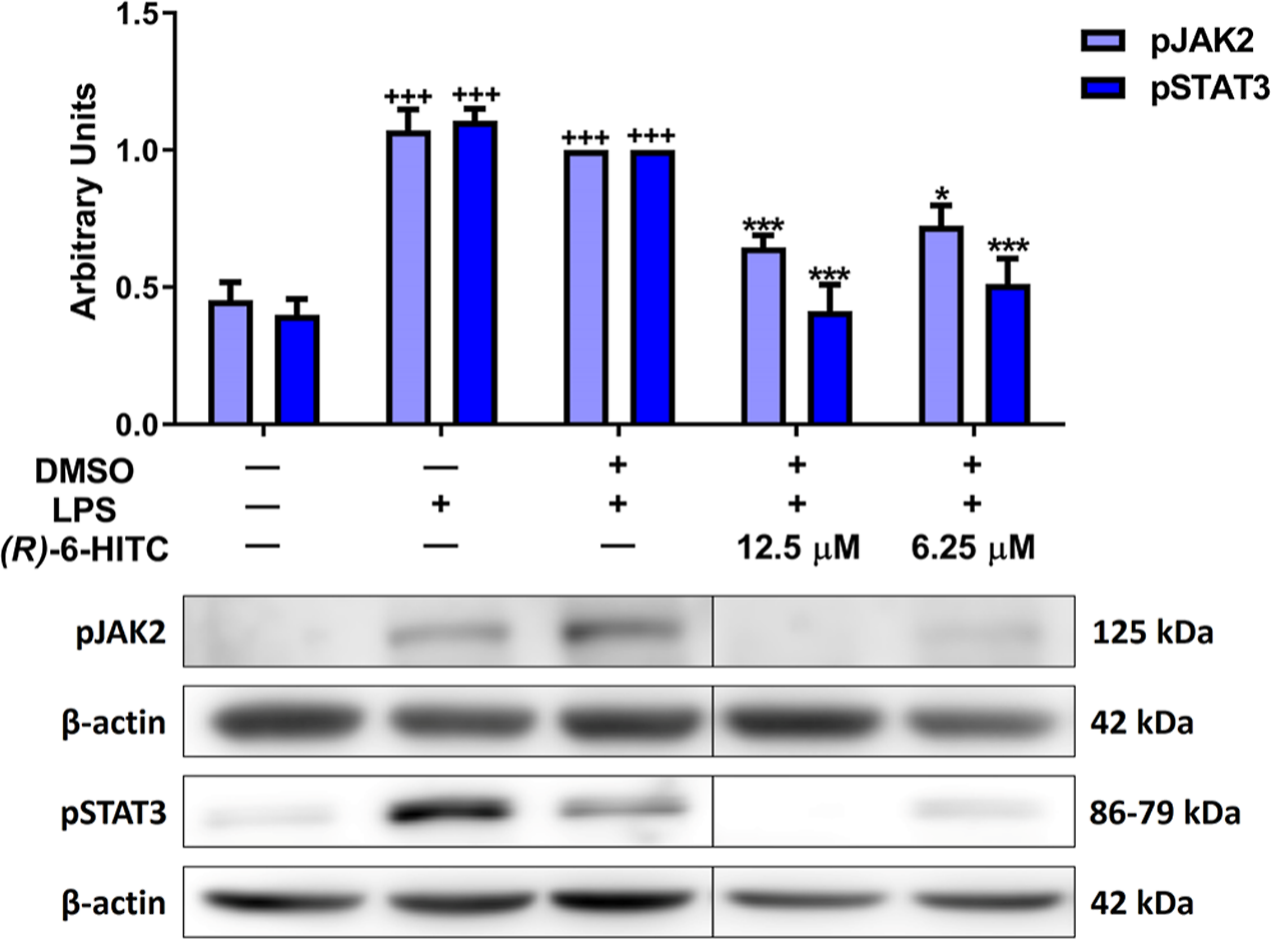
(*R*)-6-HITC reduces the phosphorylation of JAK/STAT pathway on LPS-activated peritoneal macrophages. Immune cells were stimulated with LPS (5 μg/mL) for 18 h after treating cells with (*R*)-6-HITC (12.5 and 6.25 μM) for 30 min. Histograms show the densitometric analysis of pJAK2 and pSTAT3 protein normalized to β-actin housekeeping gene. Data shown are means ± SEM (*n* = 6). (+++) *p* < 0.001 vs nonstimulated control cells; (*) *p* < 0.05; (***) *p* < 0.001 vs LPS-DMSO-treated cells.

Due to the fact that the immune response is coordinated and mediated by soluble mediators, which are mostly pro-inflammatory cytokines, the Janus kinase/signal transducer and activator of the transcription (JAK/STAT) signaling pathway is a therapeutic target in several immune-mediated inflammatory diseases. Therefore, we evaluated the role of (*R*)-6-HITC in this pathway. From Figure 7, it can be noted that LPS stimulation in murine peritoneal macrophages produced a significant increase in the phosphorylation of JAK2 and STAT3 proteins (+++*p* < 0.001 vs unstimulated control cells), which were significantly reversed by treatment with this natural ITC (12.5 and 6.25 μM) (*p < 0.05; ***p < 0.001 vs LPS-DMSO-treated cells).

### 3.8 (*R*)-6-HITC Inhibited Canonical and Noncanonical Inflammasomes in LPS-Activated Murine Peritoneal Macrophages

To better understand the anti-inflammatory mechanisms exerted by (*R*)-6-HITC, we proceeded to explore the activity of this ITC in canonical and noncanonical signaling pathways of inflammasomes.

**Figure 8:**
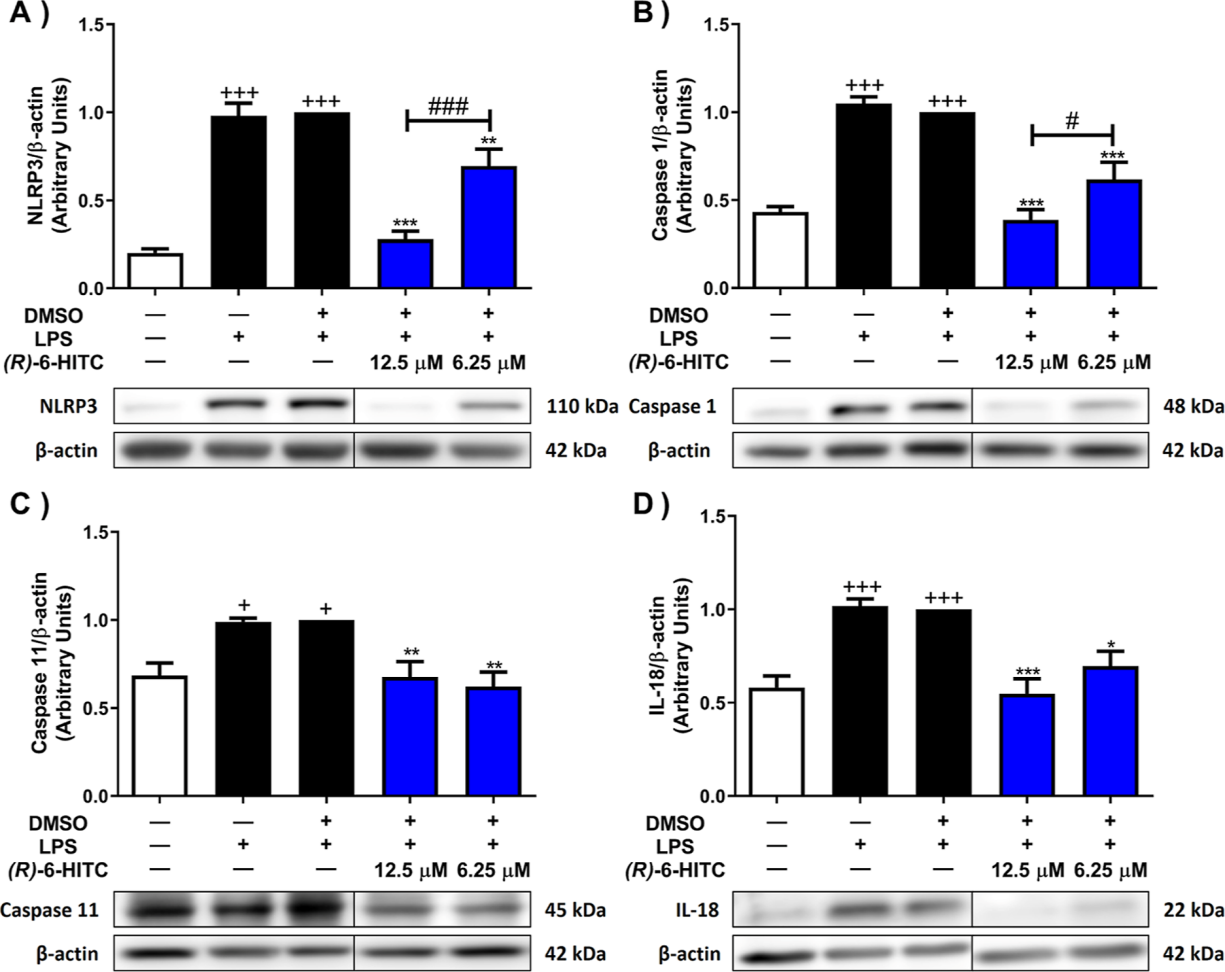
(*R*)-6-HITC inhibits canonical and noncanonical inflammasome signaling pathways in peritoneal macrophages stimulated by LPS. (*R*)-6-HITC (12.5 and 6.25 μM) was used to treat immune cells for 30 min. Later, cells were exposed to LPS for 18 h. Histograms show the densitometric analysis of NLRP3 (A), caspase 1 (B), caspase 11 (C), and IL-18 (D) proteins normalized to β-actin housekeeping gene. Data shown are means ± SEM (*n* = 6). (+) *p* < 0.05; (+++) *p* < 0.001 vs nonstimulated control cells; (*) *p* < 0.05; (**) *p* < 0.01; (***) *p* < 0.001 vs LPS-DMSO-treated cells; (#) *p* < 0.05; (###) *p* < 0.001 vs cells treated with 6.25 μM (*R*)-6-HITC.

As can be seen in Figure 8, LPS stimulation in peritoneal macrophages produced a significant upregulation of the expression of nucleotide-binding domain and leucine-rich repeat protein 3 (NLRP3), caspase 1, and caspase 11 proteins (+*p* < 0.05 and + ++*p* < 0.001 vs unstimulated control cells). Notwithstanding, the treatment with both concentrations of (*R*)-6-HITC significantly mitigated NLRP3, caspase 1, and caspase 11 (Figure 8A–C) (**p < 0.01; ***p < 0.001 vs LPS-DMSO-treated cells) protein expression. Interestingly, the highest concentration of this ITC was able to significantly reduce NLRP3 and caspase 1 expression even more than the lowest concentration (Figure 8A,B) (#*p* < 0.05; ###*p* < 0,001 vs cells treated with 6.25 μM (*R*)-6-HITC).

The activation of the NLRP3 inflammasome through the canonical and noncanonical pathways leads to the maturation of the pro-IL-1β and pro-IL-18 cytokines to IL-1β and IL-18^33^, respectively. Thus, we also determined IL-1β (Figure 2A) and IL-18 (Figure 8D) cytokine production by ELISA and Western blot assays, respectively. Both cytokines were significantly overproduced in macrophages when they were stimulated by LPS (+++ *p* < 0.001 vs control cells unstimulated). However, 12.5 and 6.25 μM of this compound significantly down-regulated the production of both pro-inflammatory markers (*p < 0.05; ***p < 0.001 vs LPS-DMSO-treated cells) (Figure 3A,D, respectively).

## 4 Discussion

Our results have shown for the first time that (*R*)-6-HITC exhibits immunomodulatory effects in LPS-stimulated murine peritoneal macrophages.

LPS-induced activation is associated with an imbalance of the cytokine network, leading to increased expression of several pro-inflammatory cytokines such as IL-17, TNF-α, IL-1β, and IL-6.^34−36^ In line with this scientific evidence, our results have shown that LPS significantly increased the levels of these pro-inflammatory cytokines. However, (*R*)-6-HITC pretreatment modulated the expression of these cytokines, counteracting the LPS effect. Analogous results have been obtained with other natural ITCs including benzyl ITC (BITC) in RAW 264.7 cells^37^ or (*R*)-SFN in murine peritoneal macrophages.^27^

Activation of the COX-2/mPGES-1 axis is an additional outcome of LPS-induced stimulation in peritoneal macrophages. When LPS binds to toll-like receptor (TLR)-4, it triggers the activation of phospholipase A2 (PLA2), leading to the conversion of membrane phospholipids to arachidonic acid (AA), the precursor of prostaglandin E2 (PGE2). Subsequently, AA is transformed into PGH2 by COX-2, which is used by mPGES-1 to generate PGE2.^38,39^ In agreement with previous studies, our findings revealed that LPS stimulation resulted in increased expression of the pro-inflammatory enzymes COX-2 and mPGES-1. However, pretreatment with (*R*)-6-HITC was able to modulate their overexpression, representing an interesting molecular target for this ITC, which has not been previously described. Similar results have been obtained with other ITCs like berteroin in the RAW 264.7 macrophage cell line.^40^

Excessive ROS production is associated with detrimental effects. These include the production and release of inflammatory mediators as well as a decrease in the ability of the enzymatic antioxidant system to counteract it. Several sources, including the mitochondrial, such as the electron transport chain, monoamine oxidase (MAO), or the adaptor protein P66shC, can contribute to increased ROS production.^41^ According to our results, (*R*)-6-HITC was able to effectively reduce the levels of intracellular ROS production generated by the action of bacterial LPS. Our data also agree with moringa ITC-1 (MIC-1) in the RAW 264.7 macrophage line.^42^

INOS is acknowledged for its pro-inflammatory and pro-oxidative properties and facilitates the synthesis of NO through the conversion of l-arginine into NO, with the assistance of oxygen and electrons provided by NADPH.^43,44^ In the presence of LPS, iNOS is upregulated, leading to increased levels of NO, which serves as a critical regulator of immune system functionality, modulating intracellular ROS production and thereby contributing to redox disbalance.^45^

Accordingly, an increase in iNOS enzyme expression and levels of nitrite, a stable product of NO, were observed in the stimulated groups. In contrast, these effects were mitigated by pretreatment with (*R*)-6-HITC. Comparable results were observed with other ITCs including SFN in an in vitro model of murine peritoneal macrophages^46^ and allyl ITC (AITC) in the BV2 cell line.^47^

Nrf2 is a key transcription factor in cellular antioxidant defense. It is inactivated in the cytoplasm under basal conditions by Kelch-like ECH-associated protein 1 (Keap1). In situations of oxidative stress, where there is a high proportion of electrophilic and oxidative compounds, activation of the Keap1/Nrf2 system occurs. These electrophiles react with cysteines reactive to the repressor, producing a conformational change in its structure. Then, Nrf2 is released for its future translocation to the nucleus, where it will bind to a specific area of DNA, known as antioxidant response elements, responsible for the gene expression of different antioxidant enzymes, such as HO-1.^48^

Several studies have been conducted regarding the activity of ITCs on the Nrf2/HO-1 axis, which include compounds such as SFN, iberverin, cheirolin, and iberin, indicating their potential to induce this pathway.^49,50^ Hence, the influence of (*R*)-6-HITC on the Nrf2/HO-1 axis was also investigated. The pretreatment with (*R*)-6-HITC induced the protein expression of Nrf2 and consequently HO-1, which may be responsible for its antioxidant activity. Other ITCs have shown similar activities like phenethyl ITC (PEITC), (*R*)-SFN, and (*R*)-8-methylsulfinyloctyl ITC [(*R*)-8-OITC] in peritoneal macrophages^27,32,51^ and moringin in the RAW 264.7 cell line.^52^

MAPKs are composed of three families of proteins: p38, ERKs, and JNKs. These proteins act as key signaling proteins in response to a wide variety of stimuli, for instance, stress or the presence of cytokines. Several processes including cell death, differentiation, homeostasis, and proliferation are regulated by this set of proteins.^53^ Scientific evidence suggests that LPS stimulation of mouse peritoneal macrophages resulted in phosphorylation of p38, JNK, and ERK1/2 MAPKs.^27,45^ Consistent with the literature, a significant increase in MAPKs phosphorylation was produced; however, (*R*)-6-HITC pretreatment significantly downregulated MAPKs activation. AITC and BITC have shown similar activity in the AGS and THP-1 cell lines, respectively.^54,55^

We also studied the JAK/STAT signaling pathway that regulates inflammatory response. There are various families of receptors that operate through this route to transduce signals in response to specific stimuli such as cytokines or growth factors. JAKs are cytoplasmic proteins that associate with receptors via their intracellular tails. Upon binding of the ligand to the receptor, the spatial conformational change of JAKs is triggered, resulting in their activation by selective phosphorylation of tyrosine residues. The receptor cytoplasmic region is phosphorylated through the tyrosine residues of activated JAKs, which create binding sites for STATs. After binding, STATs are phosphorylated and subsequently form dimers translocating to the cell nucleus, regulating the gene transcription responsible for regulating cellular processes such as apoptosis, proliferation, and differentiation.^56^

In previous studies,^27,57^ macrophage exposure to LPS resulted in an increase in the phosphorylation levels of JAK2 and STAT3 proteins. Nonetheless, (*R*)-6-HITC was able to downregulate the phosphorylation for both proteins. Similar results were observed with the *R* enantiomer of SFN and 8-OITC in murine peritoneal activated by LPS^27,32^ and PEITC, respectively, which could inhibit STAT3 activation in the DU145 cell line.^58^

An important component of the innate immune system is the inflammasome, the most well-known being the NLRP3 inflammasome. It can be activated through two pathways: canonical and noncanonical. This multiprotein complex is formed by NLRP3, the caspase recruitment domain (ASC), and caspase 1. To become activated, it requires two signals: an initial one, such as LPS binding to TLR-4, which induces the protein expression of NLRP3, pro-IL-1β, and pro-IL-18, and a second signal, such as ATP or crystalline substances, for assembly and activation. It consists of the recruitment of ASC by NLRP3 and its subsequent binding to pro-caspase 1 to form the complex with activated caspase 1, leading to the maturation of the pro-inflammatory cytokines IL-1β and IL-18. On the noncanonical pathway, LPS activation at TLR-4 causes translocation of nuclear factor-κB (NF-κB) to the nucleus, activating the expression of genes encoding inflammatory proteins, including NLRP3, IL-1β, and IL-18. Moreover, caspase 11 is upregulated through the TRIF-mediated JAK/STAT pathway, triggering pyroptosis by the activation of the noncanonical inflammasome pathway.^59^

On the basis described above, LPS-stimulated macrophages had increased protein expression of NLRP3, caspase 1, caspase 11, IL-1 β, and IL-18. In contrast, immune cells treated with (*R*)-6-HITC significantly reduced their levels of protein expression. Thus, we provide evidence confirming that (*R*)-6-HITC modulated inflammatory responses by inhibiting the NLRP3 inflammasome via caspase 1 and caspase 11. However, BITC and berteroin have shown similar activity in the inflammasome in primary Kuppfer cells and in primary bone marrow-derived macrophage cells, respectively,^60,61^ as well as (*R*)-SFN and (*R*)-8-OITC in murine peritoneal activated by LPS.^27,32^

Collectively, our data confirmed for the first time the role of (*R*)-6-HITC, the major ITC of wasabi, as a modulator of the inflammatory response and oxidative stress induced by LPS stimulation in mouse peritoneal macrophages, reducing pro-inflammatory enzyme expressions (COX-2, mPGES-1, and iNOS) and cytokine production (IL-1β, IL-6, IL-17, IL-18, and TNF-α) by inhibiting key signaling pathways such as MAPKs (ERK, JNK, and p38), JAK2/STAT3 and both canonical and noncanonical inflammasome pathways. Furthermore, the compound could reduce nitrite and ROS levels and upregulate the Nrf2/HO-1 axis in these immune cells. Consequently, (*R*)-6-HITC could be a new nutraceutical compound useful for immunoinflammatory disease management. Although these results are very promising, it must be emphasized that further in vivo investigations are needed to fully explore the immunomodulatory potential of (*R*)-6-HITC.

## Supporting Information Available

^1^H NMR and ^13^C NMR of 6-azidohexan-1-ol (**1**); ^1^H NMR and ^13^C NMR 6-azidohexyl methanesulfonate (**2**); ^1^H NMR and ^13^C NMR of 6-azidohexyl-1-thioacetate (**3**); ^1^H NMR of 6-azidohexane-1-sulfinyl chloride (**4**); ^1^H NMR and ^13^C NMR of (*S*)-(1,2:5,6-di-*O*-isopropylidene-α-d-glucofuranosyl) 6-azidohexanesulfinate (**5-(*S*)**); ^1^H NMR and ^13^C NMR of (*R*)-(−)-1-azido-6-(methylsulfinyl)-hexane (**6-(*R*)**); ^1^H NMR and ^13^C NMR of (*R*)-(−)-1-isothiocyanato-6-(methylsulfinyl)-hexane (**(*R*)-6-HITC**); and HPLC chromatogram of the racemic form of 6-HITC and its enantiopure form **(*R*)-6-HITC** (PDF)

## Abbreviations

AA: arachidonic acid
AITC: allyl isothiocyanate
ARE: antioxidant response elements
ASC: caspase recruitment domain
BEITC: benzyl isothiocyanate
COX-2: cyclooxygenase-2
DCFDA: 2′ 7′-dichlorofluorescin-diacetate
DSS: dextran sulfate sodium
ERK: extracellular signal-regulated kinase
FCS: fetal calf serum
GLS: glucosinolate
HO-1: heme oxygenase-1
HPLC: high-performance liquid chromatography
HRMS: high-resolution mass spectra
IL: interleukin
iNOS: inducible nitric oxide synthase
ITC: isothiocyanate
JAK: Janus kinase
JNK: c-Jun N-terminal kinase
Keap1: Kelch-like ECH-associated protein 1
LPS: lipopolysaccharide
MAO: monoamine oxidase
MAPK: mitogen-activated protein kinase
MIC-1: moringa isothiocyanate-1
mPGES-1: microsomal prostaglandin E synthase-1
NF-κB: nuclear factor-κB
NLRP3: nucleotide-binding domain and leucine-rich repeat protein 3
NMR: nuclear magnetic resonance
NO: nitric oxide
Nrf2: nuclear factor erythroid 2-related factor 2
PBS: phosphate buffer saline solution
PEITC: phenethyl isothiocyanate
PGE2: prostaglandin E2
PLA2: phospholipase A2
ROS: reactive oxygen species
SEM: standard error of the mean
SFN: sulforaphane
SRB: sulforhodamine B
STAT: signal transducer and activator of transcription
TLR: toll-like receptor
TNF: tumor necrosis factor
(*R*)-6-HITC: (*R*)-(−)-1-isothiocyanato-6-(methylsulfinyl)-hexane
(*R*)-8-OITC: (*R*)-8-methylsulfinyloctyl isothiocyanate
6-HITC: 6-methylsulfinylhexyl isothiocyanate
