# Supplementary material for: Immunomodulatory Effects and Regulatory Mechanisms of (R)-6-HITC, an Isothiocyanate from Wasabi (Eutrema japonicum), in an Ex Vivo Mouse Model of LPS-Induced Inflammation
Source: J Agric Food Chem. 2024 Sep 19;72(39):21520–32. doi: 10.1021/acs.jafc.4c02943 (PMC11450934; doi:10.1021/acs.jafc.4c02943)
Supplement: Supplementary file 1 — jf4c02943_si_001.pdf [file jf4c02943_si_001.pdf]

Supporting Information

Immunomodulatory Effects and Regulatory Mechanisms of (*R*)-6-HITC, an Isothiocyanate from Wasabi (*Eutrema japonicum*), in an *Ex Vivo* Mouse Model of LPS-induced Inflammation

Manuel Alcarranza[a,b*], Catalina Alarcón-de-la-Lastra[a,b], Rocío Recio Jiménez[c], Inmaculada Fernández[c], María Luisa Castejón Martínez[a,b] and Isabel Villegas[a,b*]

[a]*Instituto de Biomedicina de Sevilla, IBiS/Hospital Universitario Virgen del Rocío/CSIC/Universidad de Sevilla, 41013, Sevilla, Spain.*

[b]*Departamento de Farmacología, Facultad de Farmacia, Universidad de Sevilla, 41012 Sevilla, España.*

[c]*Departamento de Química Orgánica y Farmacéutica, Facultad de Farmacia, Universidad de Sevilla, 41012 Sevilla, España.*

[*]Email: malcarranza@us.es; Phone: +34 954556503

[*]Email: villegas@us.es; Phone: +34 954556503

List of supplementary data Pag.

**Figure S1**. ^{1}H-NMR (500 MHz, $CDCl_3$) of 6-Azidohexan-1-ol (**1**).	**S3**
**Figure S2**. ^{13}C-NMR (125 MHz, $CDCl_3$) of 6-Azidohexan-1-ol (**1**).	**S3**
**Figure S3**. ^{1}H-NMR (500 MHz, $CDCl_3$) of 6-Azidohexyl methanesulfonate (**2**).	**S4**
**Figure S4**. ^{13}C-NMR (125 MHz, $CDCl_3$) 6-Azidohexyl methanesulfonate (**2**).	**S4**
**Figure S5**. ^{1}H-NMR (500 MHz, $CDCl_3$) of 6-Azidohexyl-1-thioacetate (**3**).	**S5**
**Figure S6**. ^{13}C-NMR (125 MHz, $CDCl_3$) of 6-Azidohexyl-1-thioacetate (**3**).	**S5**
**Figure S7.** ^{1}H-NMR (300 MHz, $CDCl_3$) of 6-Azidohexane-1-sulfinyl chloride (**4**).	**S6**
**Figure S8**. ^{1}H-NMR (300 MHz, $CDCl_3$) of (*S*)-(1,2:5,6-Di-*O*-isopropylidene-α-D-glucofuranosyl) 6-azidohexanesulfinate (**5-(*S*)**).	**S6**
**Figure S9**. ^{13}C-NMR (75 MHz, $CDCl_3$) of (*S*)-(1,2:5,6-Di-*O*-isopropylidene-α-D-glucofuranosyl) 6-azidohexanesulfinate (**5-(*S*)**).	**S7**
**Figure S10**. ^{1}H-NMR (500 MHz, $CDCl_3$) of (*R*)-(-)-1-Azido-6-(methylsulfinyl)-hexane (**6-(*R*)**).	**S7**
**Figure S11**. ^{13}C-NMR (125 MHz, $CDCl_3$) of (*R*)-(-)-1-Azido-6-(methylsulfinyl)-hexane (**6-(*R*)**).	**S8**
**Figure S12**. ^{1}H-NMR (500 MHz, $CDCl_3$) of (*R*)-(-)-1-Isothiocyanato-6-(methylsulfinyl)-hexane (**(*R*)-6-HITC**).	**S8**
**Figure S13**. ^{13}C-NMR (125 MHz, $CDCl_3$) of (*R*)-(-)-1-Isothiocyanato-6-(methylsulfinyl)-hexane (**(*R*)-6-HITC**).	**S9**
**Figure S14**. HPLC chromatogram of the racemic form of 6-HITC and its enantiopure form **(*R*)-6-HITC.**	**S9**

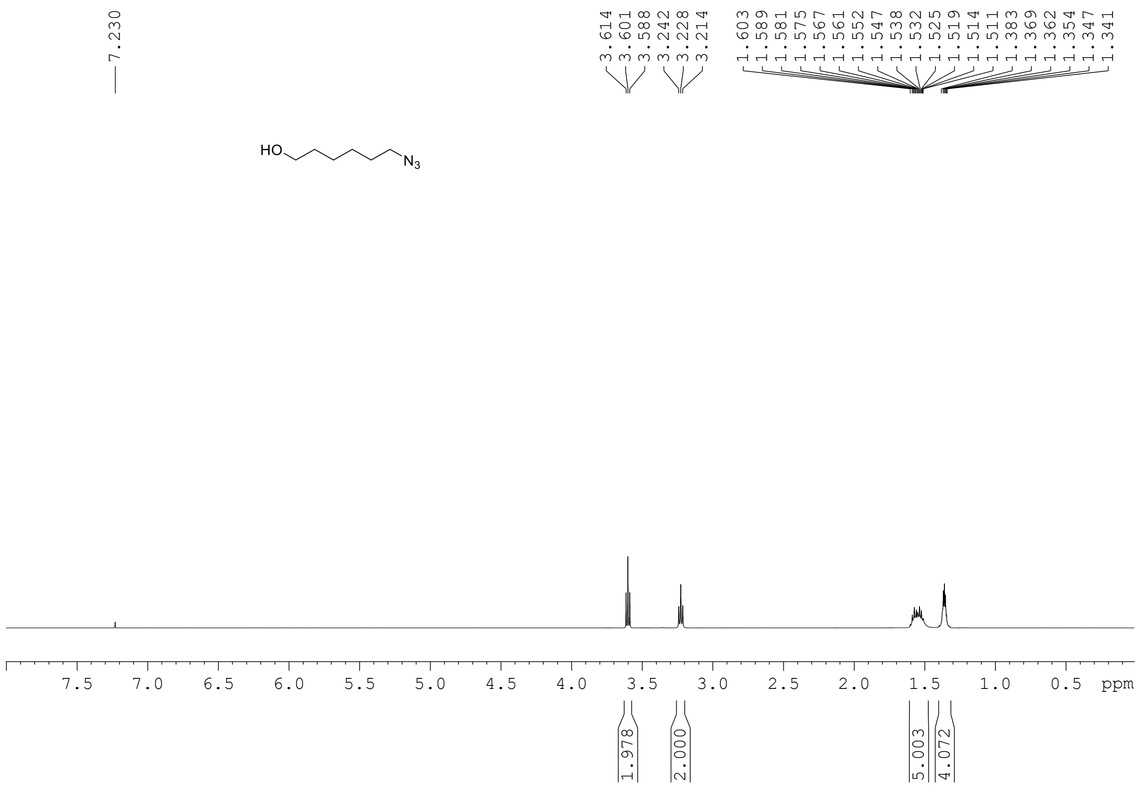

Figure S1. ^{1}H-NMR (500 MHz, $CDCl_3$) of 6-Azidohexan-1-ol (**1**).

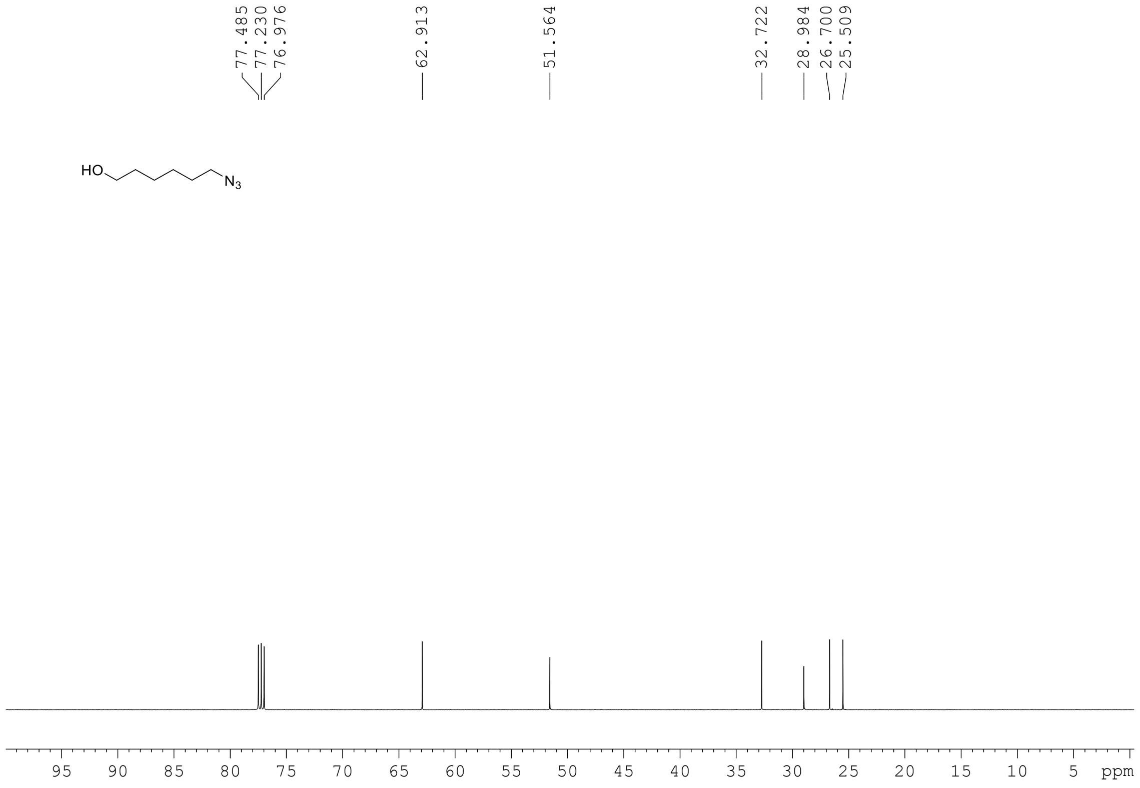

Figure S2. ^{13}C-NMR (125 MHz, $CDCl_3$) of 6-Azidohexan-1-ol (**1**).

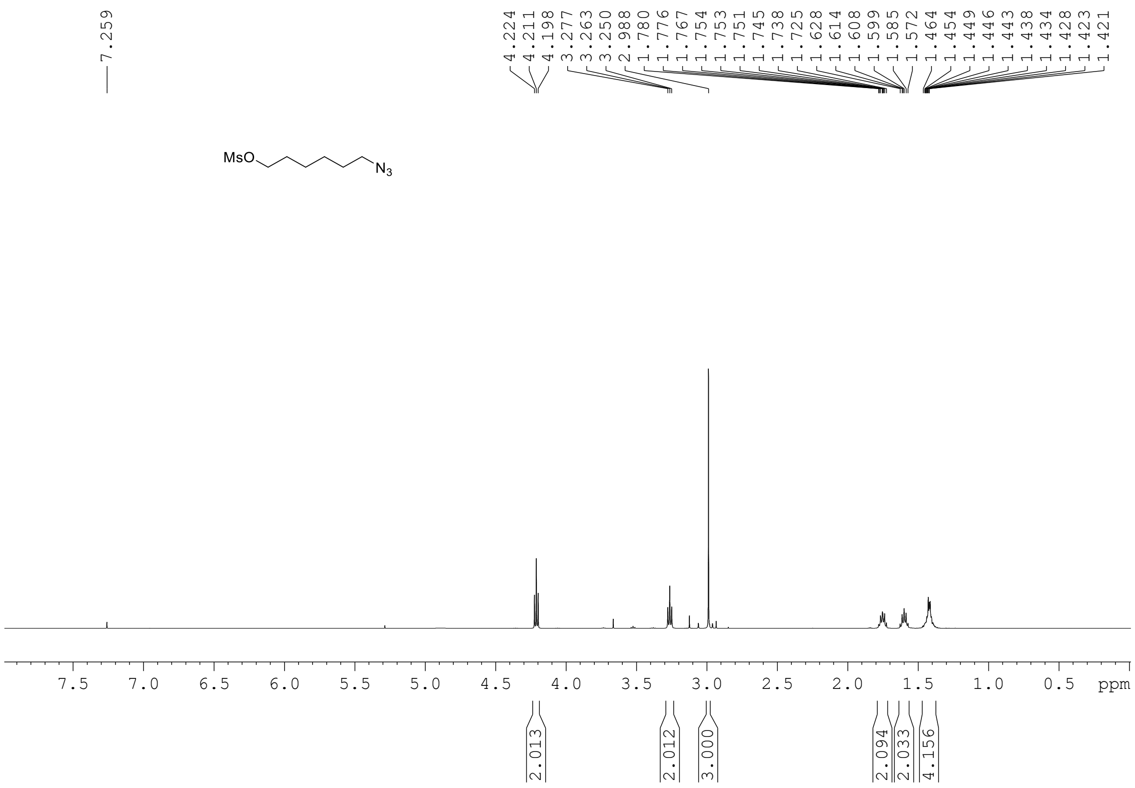

Figure S3. ^{1}H-NMR (500 MHz, $CDCl_3$) of 6-Azidohexyl methanesulfonate (**2**).

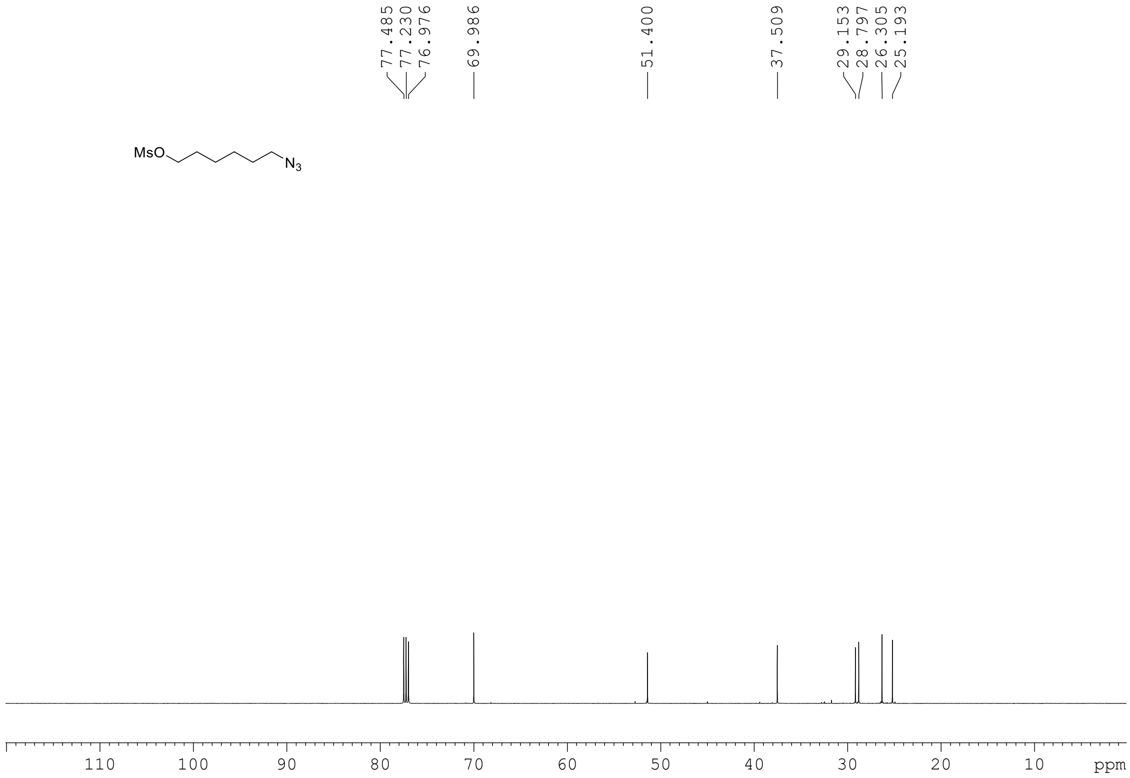

Figure S4. ^{13}C-NMR (125 MHz, $CDCl_3$) 6-Azidohexyl methanesulfonate (**2**).

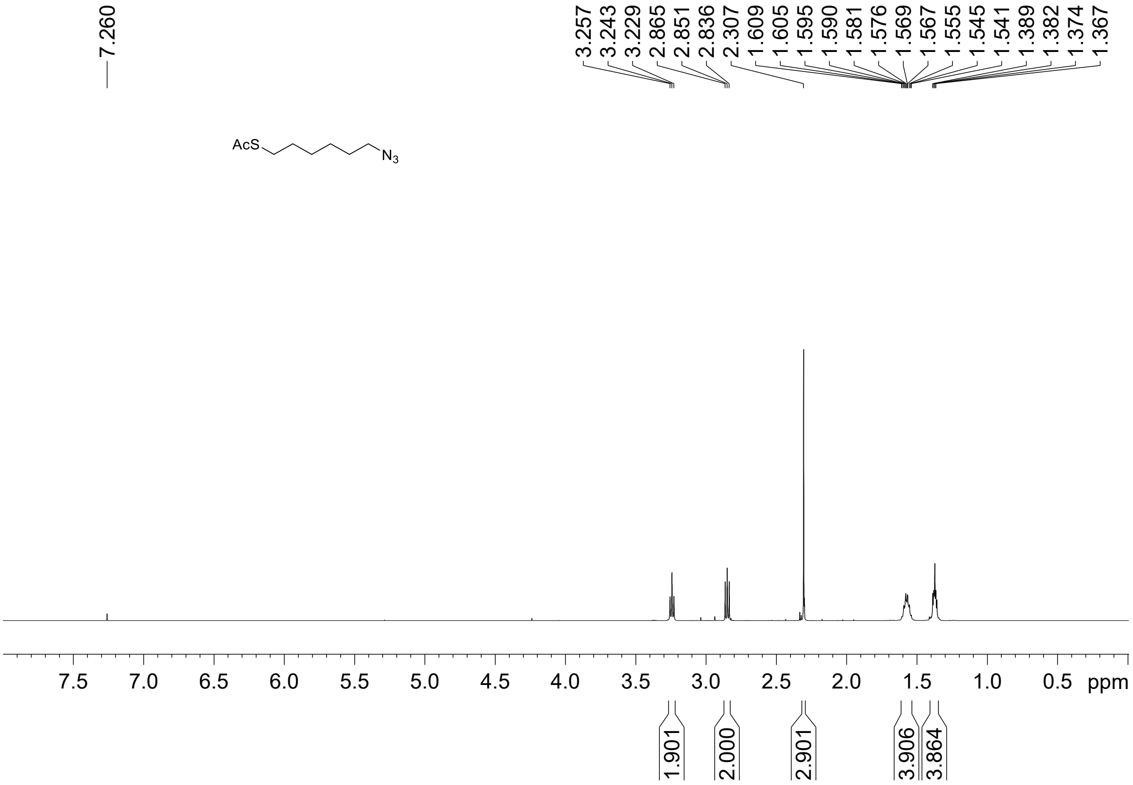

Figure S5. ^{1}H-NMR (500 MHz, $CDCl_3$) of 6-Azidohexyl-1-thioacetate (**3**).

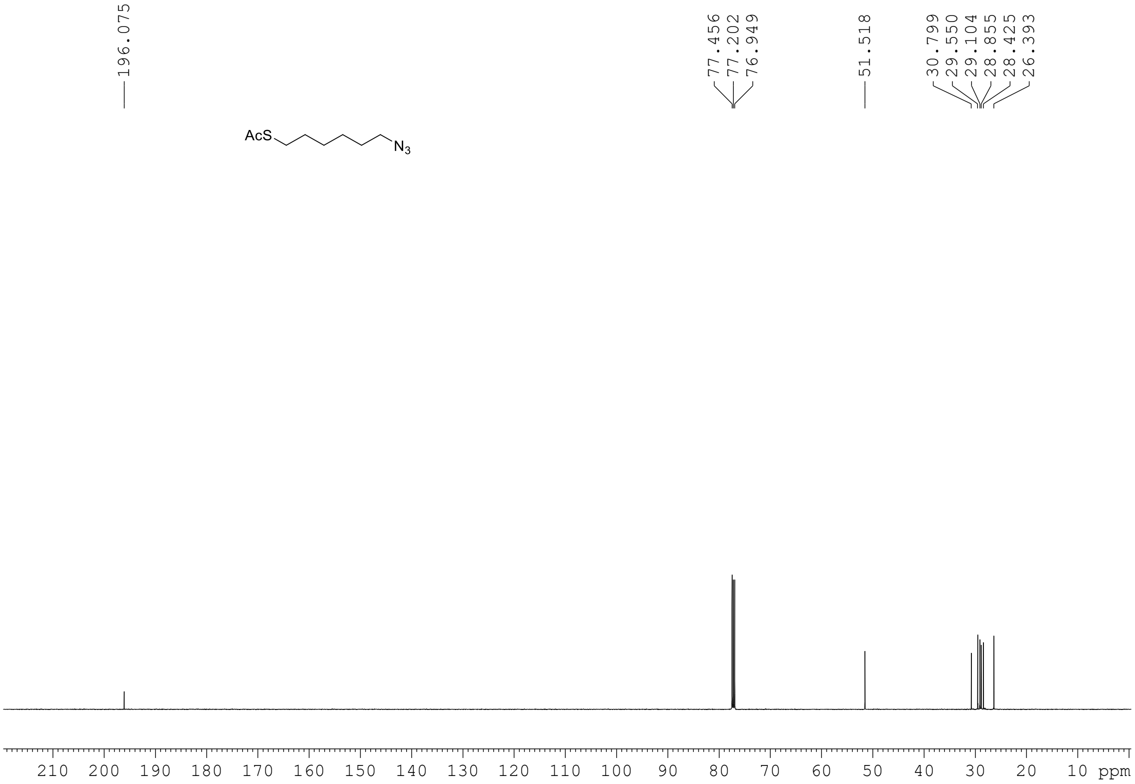

Figure S6. ^{13}C-NMR (125 MHz, $CDCl_3$) of 6-Azidohexyl-1-thioacetate (**3**).

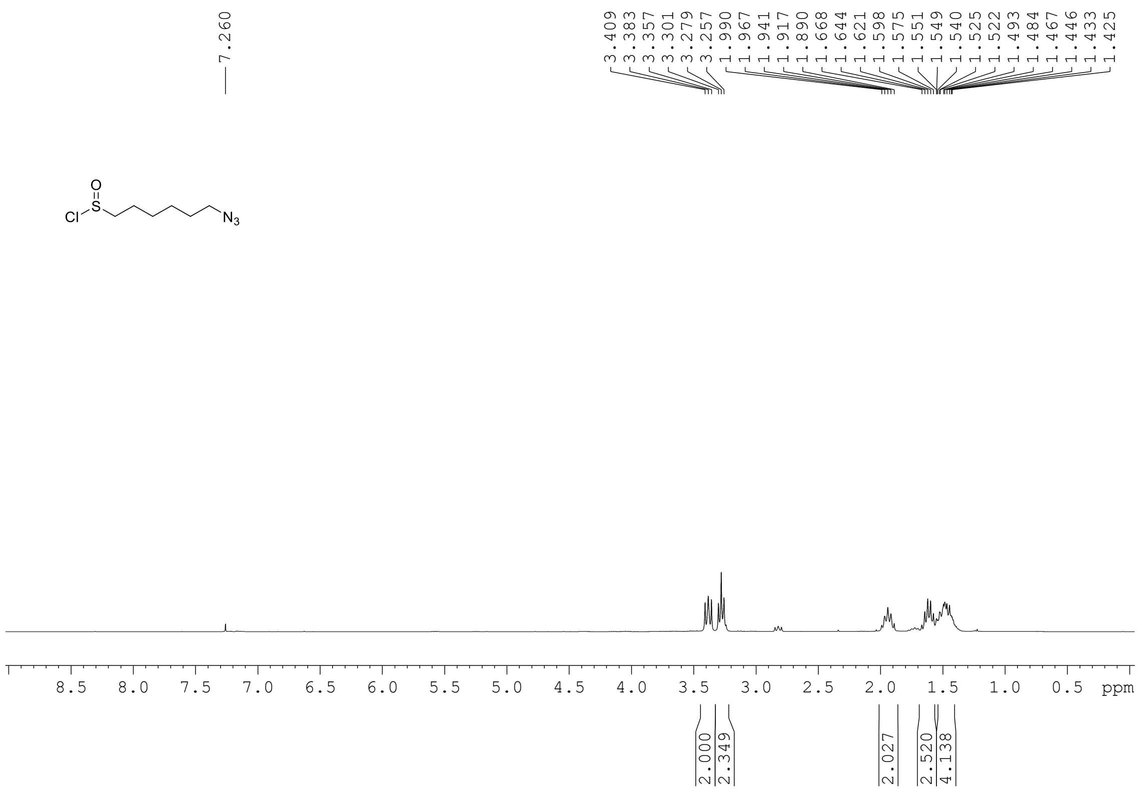

Figure S7. ^{1}H-NMR (300 MHz, $CDCl_3$) of 6-Azidohexane-1-sulfinyl chloride (**4**).

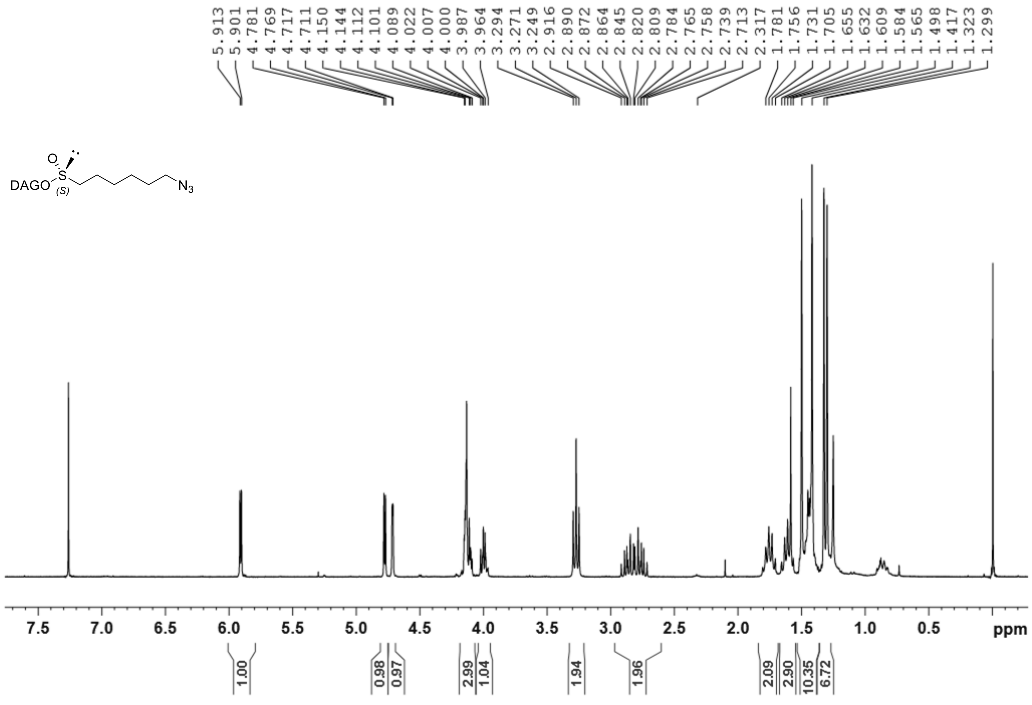

Figure S8. ^{1}H-NMR (300 MHz, $CDCl_3$) of (*S*)-(1,2:5,6-Di-*O*-isopropylidene-α-D-glucofuranosyl) 6-azidohexanesulfinate (**5-(*S*)**).

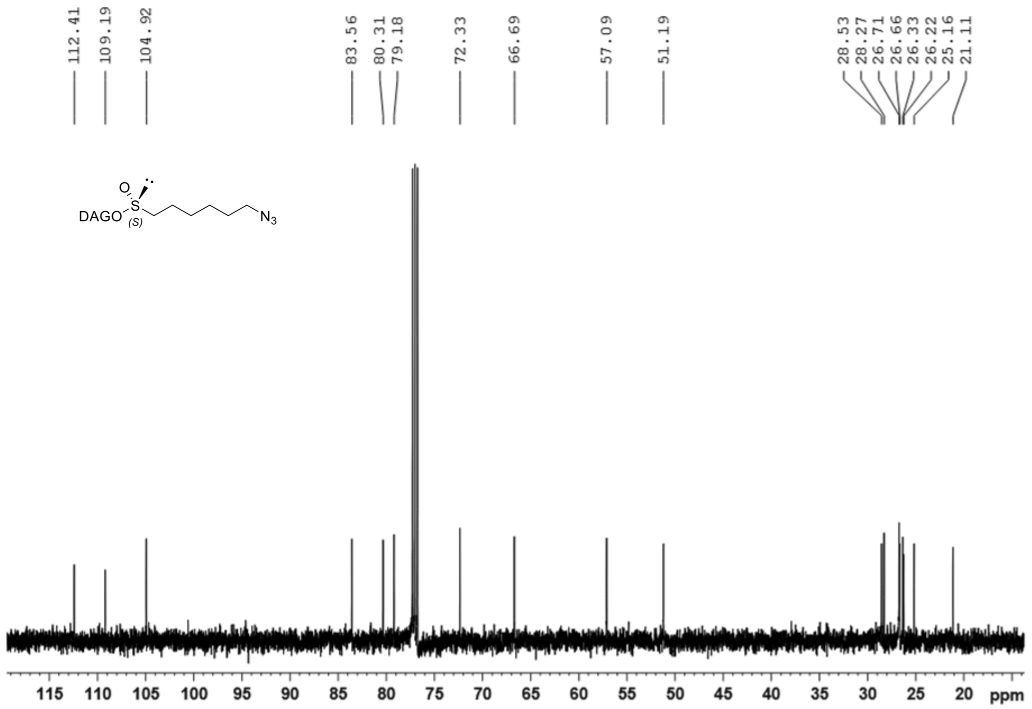

Figure S9. ^{13}C-NMR (75 MHz, $CDCl_3$) of (*S*)-(1,2:5,6-Di-*O*-isopropylidene-α-D-glucofuranosyl) 6-azidohexanesulfinate (**5-(*S*)**).

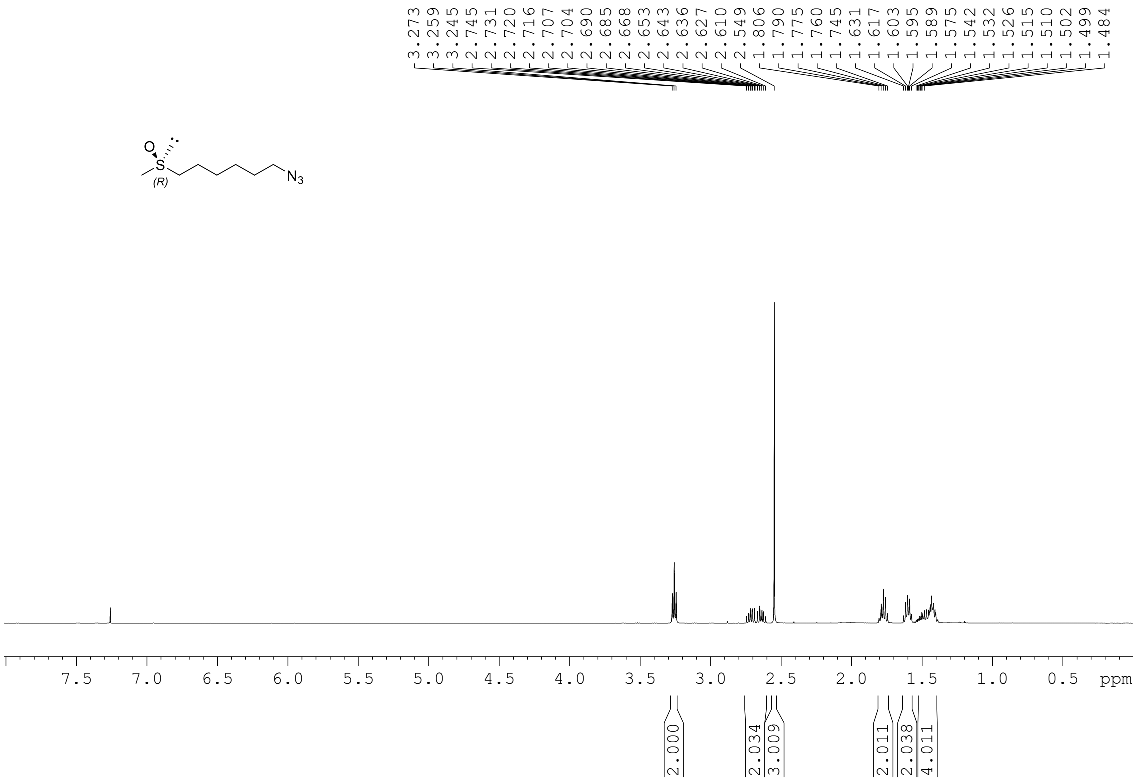

Figure S10.^{1}H-NMR (500 MHz, $CDCl_3$) of (*R*)-(-)-1-Azido-6-(methylsulfinyl)-hexane (**6-(*R*)**).

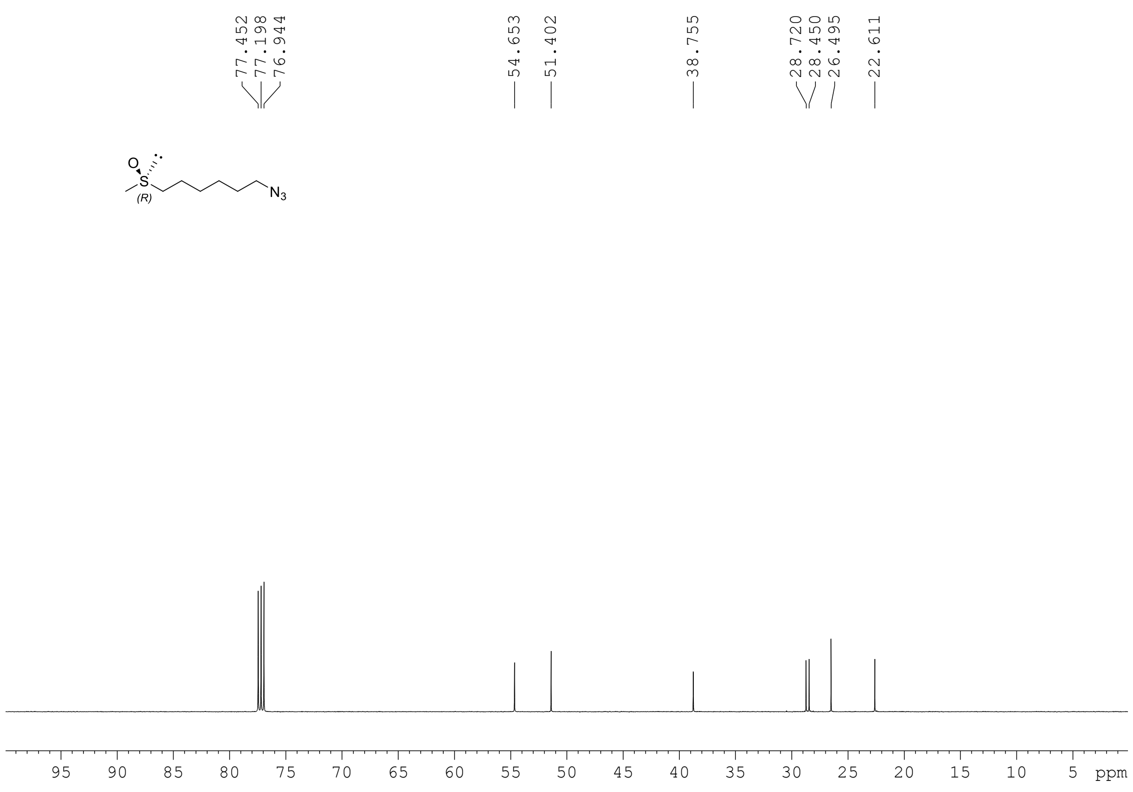

Figure S11. ^{13}C-NMR (125 MHz, $CDCl_3$) of (*R*)-(-)-1-Azido-6-(methylsulfinyl)-hexane (**6-(*R*)**).

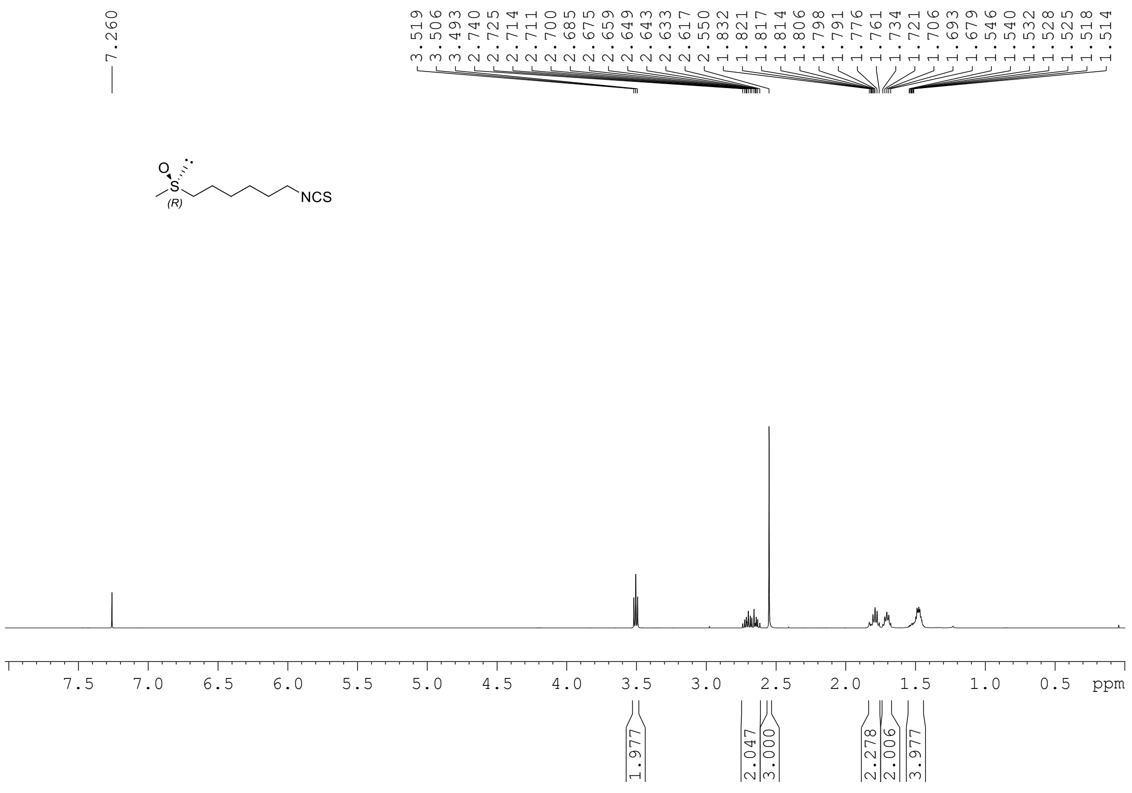

Figure S12. ^{1}H-NMR (500 MHz, $CDCl_3$) of (*R*)-(-)-1-Isothiocyanato-6-(methylsulfinyl)-hexane (**(*R*)-6-HITC**).

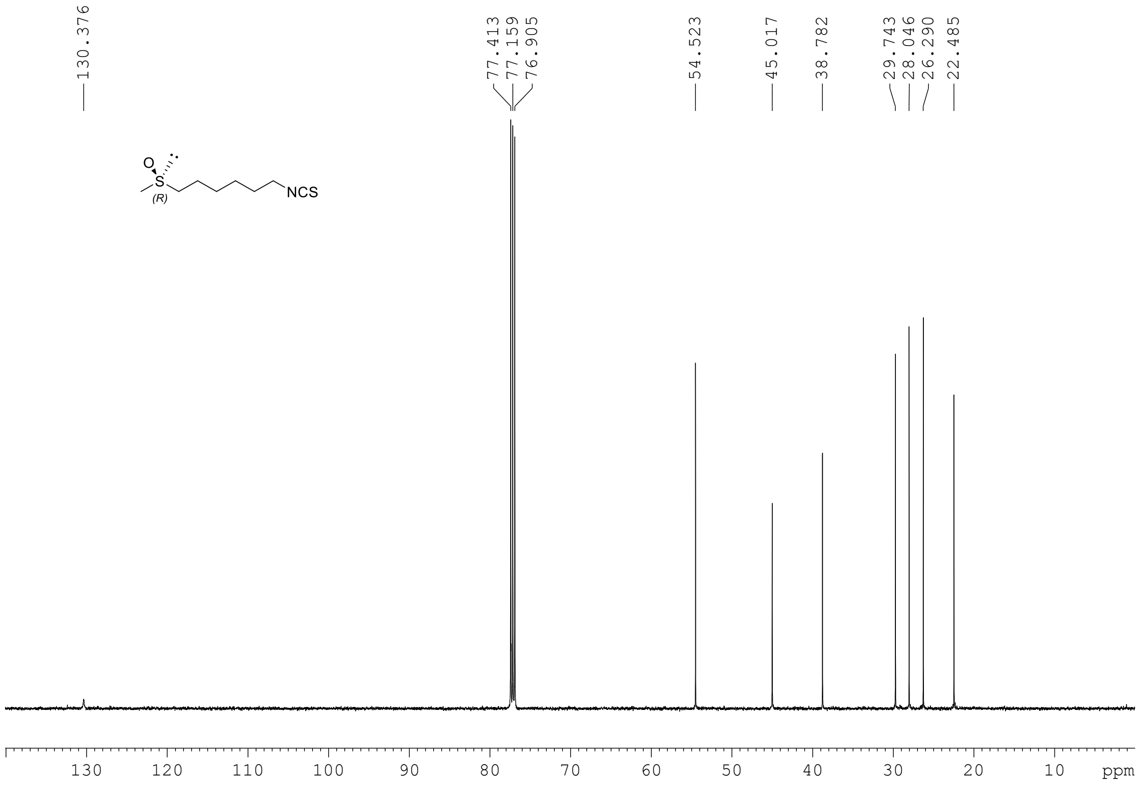

Figure S13. ^{13}C-NMR (125 MHz, $CDCl_3$) of (*R*)-(-)-1-Isothiocyanato-6-(methylsulfinyl)-hexane (**(*R*)-6-HITC**).

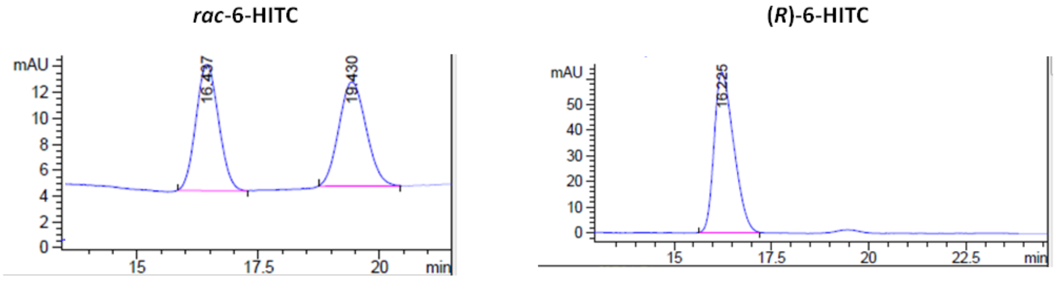

Peak #	RetTime [min]	Type	Width [min]	Area [mAU*s]	Height [mAU]	Area %
1	16.438	BB	0.4833	141.11462	4.43377	51.2339
2	19.428	BB	0.5417	134.31754	3.58029	48.7661

Peak #	RetTime [min]	Type	Width [min]	Area [mAU*s]	Height [mAU]	Area %
1	16.225	BB	0.5362	1003.66199	28.68848	100.0000

Figure S14. HPLC chromatogram of the racemic form of 6-HITC and its enantiopure form **(*R*)-6-HITC.**
